# Comparative transcriptomics of early dipteran development

**DOI:** 10.1186/1471-2164-14-123

**Published:** 2013-02-24

**Authors:** Eva Jiménez-Guri, Jaime Huerta-Cepas, Luca Cozzuto, Karl R Wotton, Hui Kang, Heinz Himmelbauer, Guglielmo Roma, Toni Gabaldón, Johannes Jaeger

**Affiliations:** 1 EMBL/CRG Research Unit in Systems Biology, Centre de Regulació Genòmica (CRG), and Universitat Pompeu Fabra (UPF), Barcelona, Spain; 2 Bioinformatics and Genomics Programme, Centre de Regulació Genòmica (CRG), and Universitat Pompeu Fabra (UPF), Barcelona, Spain; 3 CRG Bioinformatics Core, Centre de Regulació Genòmica (CRG), and Universitat Pompeu Fabra (UPF), Barcelona, Spain; 4 CRG Genomics Unit, Centre de Regulació Genòmica (CRG), and Universitat Pompeu Fabra (UPF), Barcelona, Spain; 5 Max Planck Institute for Molecular Genetics, Berlin, Germany; 6 Present address: ICFC Life Technologies, Bld #2, 218 Yindu Road, Shanghai, 200231, P. R. China; 7 Present address: Developmental and Molecular Pathways, Novartis Institute for Biomedical Research, Basel, Switzerland; 8 Centre de Regulació Genòmica (CRG), Dr. Aiguader 88, Barcelona, 08003, Spain

**Keywords:** Non-drosophilid diptera, *Clogmia albipunctata*, *Megaselia abdita*, *Episyrphus balteatus*, Comparative transcriptomics, RNA-seq, *De novo* assembly, Automated annotation, Evolutionary developmental biology, Phylogenomics

## Abstract

**Background:**

Modern sequencing technologies have massively increased the amount of data available for comparative genomics. Whole-transcriptome shotgun sequencing (RNA-seq) provides a powerful basis for comparative studies. In particular, this approach holds great promise for emerging model species in fields such as evolutionary developmental biology (evo-devo).

**Results:**

We have sequenced early embryonic transcriptomes of two non-drosophilid dipteran species: the moth midge *Clogmia albipunctata,* and the scuttle fly *Megaselia abdita*. Our analysis includes a third, published, transcriptome for the hoverfly *Episyrphus balteatus*. These emerging models for comparative developmental studies close an important phylogenetic gap between *Drosophila melanogaster* and other insect model systems. In this paper, we provide a comparative analysis of early embryonic transcriptomes across species, and use our data for a phylogenomic re-evaluation of dipteran phylogenetic relationships.

**Conclusions:**

We show how comparative transcriptomics can be used to create useful resources for evo-devo, and to investigate phylogenetic relationships. Our results demonstrate that *de novo* assembly of short (Illumina) reads yields high-quality, high-coverage transcriptomic data sets. We use these data to investigate deep dipteran phylogenetic relationships. Our results, based on a concatenation of 160 orthologous genes, provide support for the traditional view of *Clogmia* being the sister group of Brachycera (*Megaselia, Episyrphus, Drosophila*), rather than that of Culicomorpha (which includes mosquitoes and blackflies).

## Background

Comparative studies based on molecular data are not only essential to gain insights into genome evolution and species phylogeny, but also for the study of the function and evolutionary dynamics of developmental processes. Traditionally, such studies were based on the analysis of small sets of carefully selected rRNA- or protein-coding genes. More recently, larger sets of expressed sequence tags (ESTs), or high-throughput data based on whole-genome sequencing have been used for phylogenomics. Probably the best illustration of the importance and success of this approach is the establishment and elaboration of the new animal phylogeny [1-5]. In general, phylogenomic approaches have greatly improved our ability to robustly reconstruct highly resolved phylogenetic trees [4]. A relevant example in our context is the clarification of relationships between groups of holometabolan insects [6]. Here, we are using comparative transcriptomics — based on whole-transcriptome shotgun sequencing (RNA-seq), and *de novo* transcriptome assembly [7] — to examine deep phylogenetic relationships among Diptera (flies, midges, and mosquitoes). This approach provides sequence data for a large number of genes, which is not only useful for phylogenomic analyses, but also as a resource for rapid identification and cloning of genes. A couple of recent examples illustrate the potential of this approach. For instance, Hittinger et al. [8] used RNA-seq to resolve the evolutionary relationships of ten mosquito species. Moreover, Kalinka et al. [9] employed high-throughput transcriptome analyses to quantify variability in gene expression across developmental stages in different species of sequenced drosophilid fruit flies.

We are interested in extending such comparative transcriptomic analyses beyond drosophilids and mosquitoes with sequenced genomes [10-16]. Non-drosophilid dipteran species are becoming increasingly important as model systems to study the evolution of transcriptional regulation [17,18], cellular architecture [19], and a diverse range of developmental processes, such as axis specification [20-31], segment determination [22,25,27,28,30,32-38], morphogen-based spatial patterning (e.g. by BMP ligands, [39-41]), thoracic bristle patterning [42-45], and the specification of extra-embryonic tissues [46,47].

**Figure 1:**
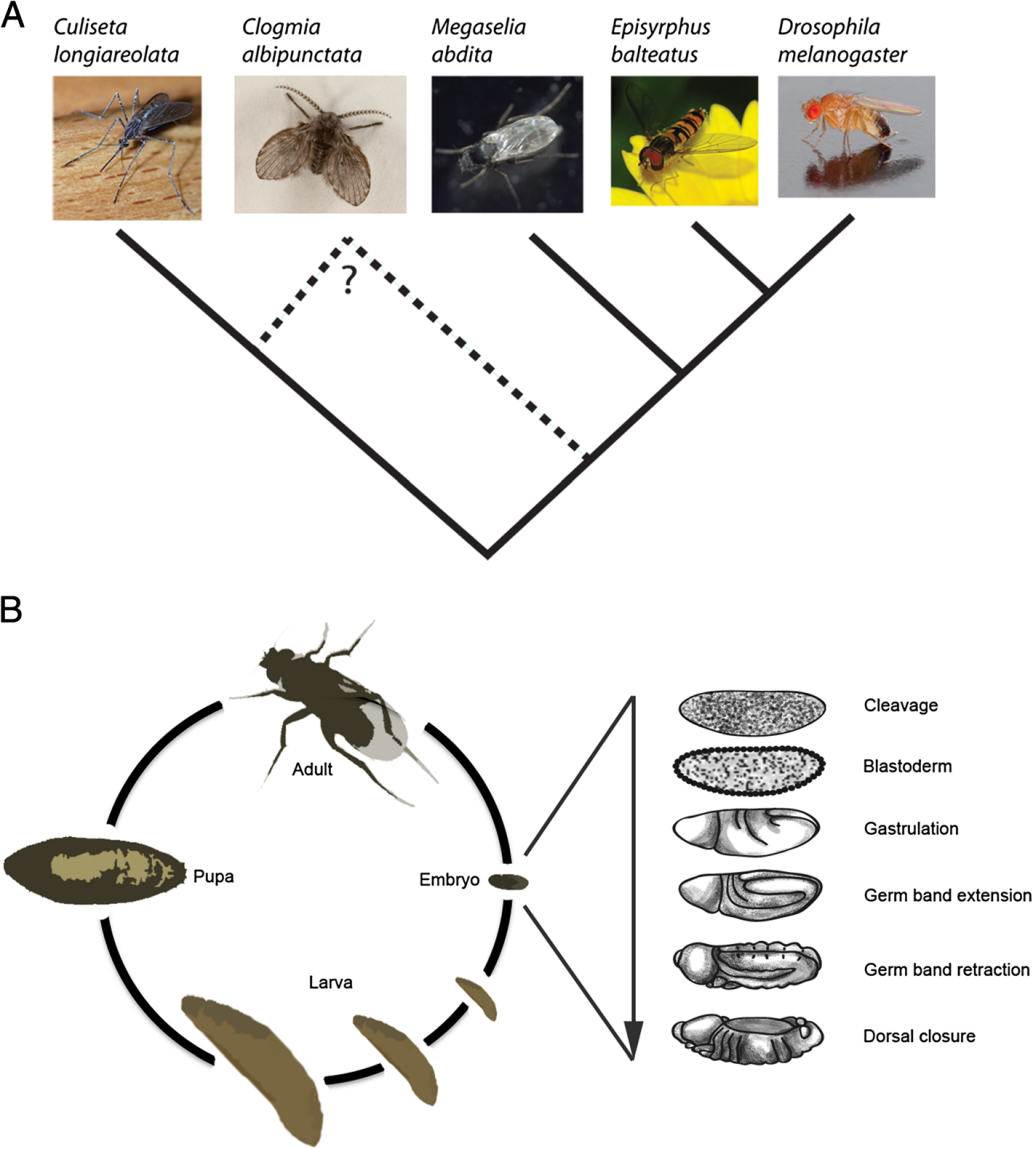
**Phylogeny and development of Diptera.** (**A**) Simplified phylogenetic tree displaying the relationships among the species used in this study, with respect to drosophilids and mosquitoes. The position of *Clogmia albipunctata* is controversial (see our Results). (**B**) Schematic representation of the dipteran life cycle, expanded view showing stages of embryo development. Transcriptomes were obtained from embryos at the following developmental stages: cleavage, blastoderm, gastrulation, and early germband extension. Image sources: *Culiseta longiareolata* and *Episyrphus balteatus* pictures by Joaquim Alves Gaspar; *Clogmia albipunctata* by Sanjay Acharya; *Drosophila melanogaster* picture by André Karwath (images publicly available through Wikimedia commons); *Megaselia abdita* picture taken by Karl R. Wotton; embryo, larvae, and fly drawings by Victor Jiménez-Guri.

Rigorous and systematic studies of the problems and processes described above require ‘omic’ resources. However, apart from three species of mosquitoes [11,14-16]—which are difficult to handle in the laboratory and to use for embryological studies—there are no published genomic data sets available for non-drosophilid dipteran species. Here, we fill this important gap by analyzing and comparing high-throughput transcriptomic data in early embryos of three emerging dipteran experimental model systems: the moth midge *Clogmia albipunctata* (family: Psychodidae)*,* the scuttle fly *Megaselia abdita* (family: Phoridae), and the hoverfly *Episyrphus balteatus* (family: Syrphidae) (Figure 1A). They were chosen based on their position in the dipteran phylogenetic tree, and their tractability for embryological studies (all of them have been established in the laboratory by Klaus Sander, Urs Schmidt-Ott, and colleagues [19,21,22,24,27,28,30,31], [34,40,41,46,47]). Of these species, only *E. balteatus* is among the 15 non-drosophilid dipterans whose transcriptomes will be sequenced as part of the 1KITE project (http://www.1kite.org), which aims at characterizing 1,000 different insects by RNA-seq.

*C. albipunctata* belongs to an early-branching dipteran lineage, which has traditionally been considered the sister group of all brachycerans (or ‘higher flies’ [48]). This position has recently been disputed, placing the psychodids as an early branch of the culicomorph lineage which includes the mosquitoes and blackflies (Figure 1A) [49]. *M. abdita* and *E. balteatus* were chosen since they belong to basally branching cyclorrhaphan lineages. The taxon Cyclorrhapha comprises the majority of brachyceran species, including the drosophilids [49]. Therefore, *M. abdita* and *E. balteatus* occupy intermediate phylogenetic positions between *C. albipunctata* and *Drosophila melanogaster* (Figure 1A)*.* In addition, *E. balteatus* is the only non-drosophilid dipteran species for which sequenced maternal and early embryonic transcriptomes are already available [40].

In this study, we used Roche 454 and Illumina HiSeq technologies and *de novo* assembly to characterize the early embryonic transcriptomes of *C. albipunctata* and *M. abdita* (Figure 1B)*.* We verify the information present in our data by manual curation and *in situ* hybridization. We compare our early embryonic transcriptomes to that of *E. balteatus*[40], as well as transcriptomic and genomic sequences from drosophilids [10,12,13,50] and/or mosquitoes [11,14,15].

Our transcriptomic data sets form the basis of a new phylogenomic assessment of gene evolutionary histories and dipteran species relationships. A Maximum Likelihood analysis of 160 concatenated orthologous genes places psychodid moth midges (such as *C. albipunctata*) as an early offshoot along the branch leading to the brachyceran lineage. This agrees with earlier morphological studies ( [48], and references therein), but stands in contrast to the recent molecular phylogeny of Wiegmann et al. [49] which places Psychodidae with mosquitos. Our analysis indicates that deep dipteran relationships remain difficult to resolve, and that more genomic and/or transcriptomic data will be needed for us to fully understand the early radiation of Diptera.

## Results and discussion

### Transcriptome sequencing, assembly, and annotation

We obtained early embryonic transcriptome sequences (covering cleavage/blastoderm stage, gastrulation, and early germband extension, Figure 1B) from the moth midge *Clogmia albipunctata* and the scuttle fly *Megaselia abdita* using RNA-seq based on the Roche 454 and Illumina HiSeq platforms (see Additional file 1, Section S1.1, for details). Raw read sequences are available from the European Nucleotide Archive (ENA) under accession number ERP001635. Our analysis also includes an early embryonic transcriptome for the hoverfly *Episyrphus balteatus*, which has been sequenced and published previously [40].

454 reads were assembled with Newbler v2.5.3 (Roche Diagnostics), while Illumina reads were assembled alone or in combination with 454 reads using the Trinity assembly tool (Version 2011-05-19 [7]; see Additional file 1, Section S1.2, for details on assembly). To compare the different assemblies and sequencing strategies, we annotated the reconstructed transcriptomes using BLASTx [51] against *Drosophila melanogaster* proteins (see Methods). Annotated transcriptome sequences are available online at http://diptex.crg.es. A detailed analysis and comparison of annotation results is presented in Additional file 1, Section S1.3.

Our analysis indicates that Illumina sequencing combined with *de novo* assembly using Trinity is a reliable approach to reconstruct transcriptomes in non-model organisms. This confirms results reported by Grabherr et al. [7]. Although 454 pyro-sequencing combined with Newbler assembly achieves longer average contig lengths, this did not result in the detection of markedly higher numbers of genes. The very extensive overlap between the different data sets indicates that we are achieving a considerable degree of saturation in our coverage.

### Verification of annotation

We assessed the quality of our transcriptome annotation by performing reciprocal BLAST searches to check for the presence or absence of 107 candidate genes known to be expressed during the blastoderm stage and early germband extension in *D. melanogaster.* The results of this analysis are summarized in Additional file 2, Section S2.1. They confirm near-saturation coverage of our data sets, and indicate that automatic pipelines lead to mis-annotation or lack of annotation for a number of genes. This number can only be reduced by careful manual curation.

**Figure 2:**
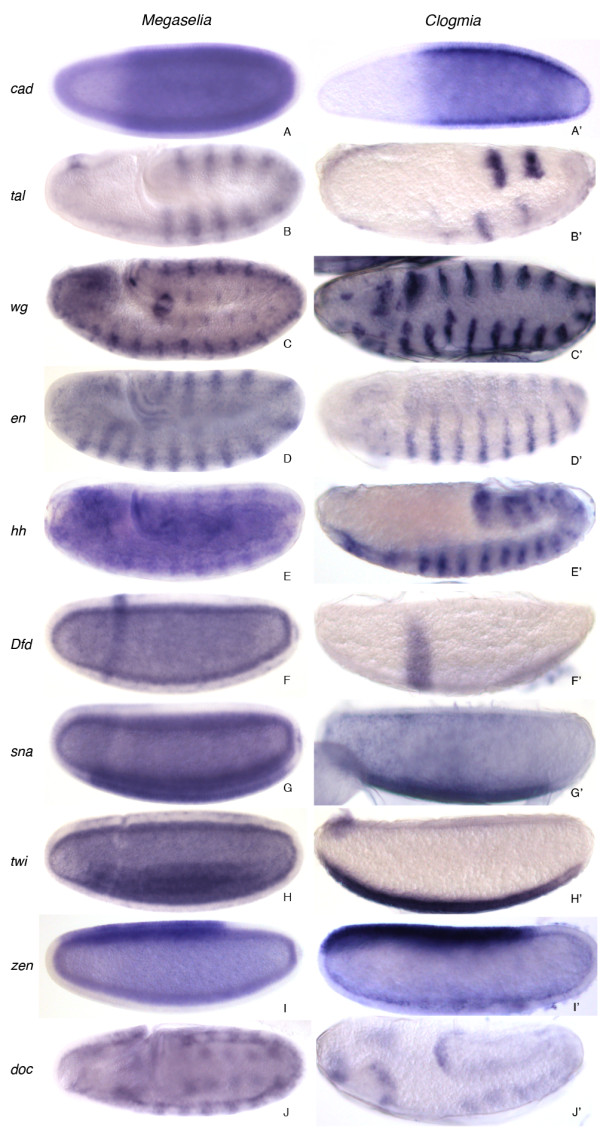
**Verification of transcriptome data by *in situ *hybridization.** We tested several selected candidate genes involved in pattern formation for spatial gene expression during the blastoderm and later stages up to the extended germband. Examples of such patterns in both *M. abdita* and *C. albipunctata* are shown. Embryos are aligned anterior to the left, dorsal up. See text for details.

Many regulatory genes expressed during early dipteran development show complex spatial expression profiles [52-55]. We used sequences present in our transcriptome data sets to make riboprobes against a set of candidate genes in order to test whether the genes present in our transcriptome data sets are expressed in spatially specific patterns between the blastoderm and the extended germband stage. Examples of conserved gene expression patterns in *M. abdita* and *C. albipunctata* are shown in Figure 2. *caudal (cad)* shows a conserved posterior expression pattern in the blastoderm as in *D. melanogaster* (Figure 2A, A'). *tarsalless (tal;* also called *mille-pattes, mlpt,* or *polished rice, pri*) is expressed in a pair-rule-like striped pattern during germband extension (Figure 2B, B'). Segment-polarity genes such as *engrailed (en), hedgehog (hh),* or *wingless (wg)* show conserved segmental pre-patterns as in *D. melanogaster* (Figure 2C–E, C'–E'). The hox gene *Deformed (Dfd)* can be detected around gastrulation time (Figure 2F, F'). Dorso-ventral and mesodermal patterning genes *twist (twi)* and *snail (sna)* show ventral expression at the blastoderm stage, and later during gastrulation (Figure 2G–H, G'–H'). *zerknüllt (zen)* is expressed at the blastoderm stage in the amnioserosa anlage (Figure 2I, I'). *dorsocross (doc)* shows a conserved expression pattern during germband extension similar to that observed in *D. melanogaster* (Figure 2J, J'). All in all, we were able to detect spatial expression (in both species) for 10 out of 17 tested candidate genes. An additional gene (*teashirt, tsh)* showed signal in *M. abdita* but not *C. albipunctata* (not shown), while the other candidates could not be cloned in either species, or did not show any consistent spatial expression patterns. This confirms the usefulness of our data sets as a resource for evolutionary developmental biology (evo-devo), since expression of genes present in our transcriptome data is also detectable by *in situ* hybridization for a majority of tested cases.

Finally, we verified our annotated data sets in terms of their ability to predict alternative splice forms. Previous work indicated that Newbler shows a low rate of false positive prediction of alternative transcripts, but fails to predict the complete set of isoforms identified by RT-PCR [56], while no equivalent evidence is available for Trinity. Our analysis (presented in Additional file 2, Section S2.2) reveals that a large percentage of the predictions by Trinity are inaccurate. Therefore, 454 pyro-sequencing and Newbler assembly should be used if reliable predictions of alternative splicing events are required.

### Comparative transcriptome analysis

Table 1 summarizes the number of genes identified by our analyses in all three species. We compare these to two estimates of the number of genes expressed during early embryogenesis in *D. melanogaster*: Lecuyer et al. [54] provide a lower limit for this number of 9,000, which is consistent with the 10,294 uniquely identified protein-coding genes present in modENCODE transcriptomes during the first four hours of development (Table 1) [50]. Our data sets contain 69.2% (*C. albipunctata*), 77.9% (*M. abdita*), and 60.2% (*E. balteatus*) of the 10,294 genes detected during early embryogenesis in *D. melanogaster*[50].

**Table 1:** Numbers of genes predicted by our analyses in each species

**Species**	**Total # of genes**
*C. albipunctata*	7,125
*M. abdita*	8,019
*E. balteatus*	6,196
*D. melanogaster*	10,294

Total number represent uniquely identified genes from both 454 (Newbler) and 454 & Illumina HiSeq (Trinity) assemblies (*C. albipunctata* and *M. abdita*), and from 454 (Newbler) and 454 (Trinity) assemblies (*E. balteatus*) taken together. The number of genes for *D. melanogaster* is determined from modENCODE RNA-seq data sets for 0–4 hrs of development; genes were considered to be expressed if RPKM>0 [50].

**Figure 3:**
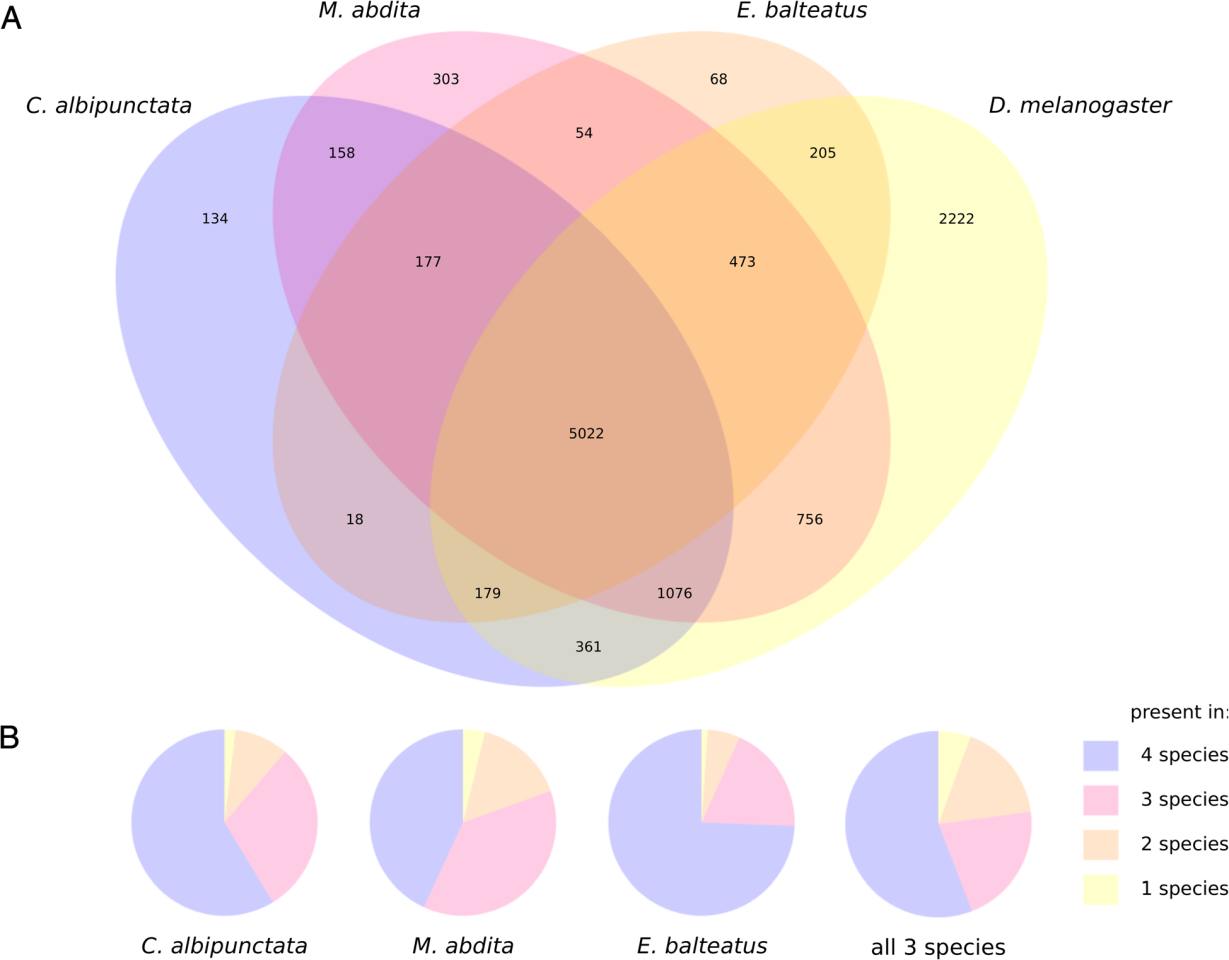
**Comparative analysis of genes detected in different species.** (**A**) Venn-diagram of annotated genes from all three species (*C. albipunctata, M. abdita,* and *E. balteatus*) compared to genes detected in the early embryonic transcriptome of *D. melanogaster* (developmental stages: 0–4 hrs, from [50]; see also Table 1). (**B**) Pie charts showing the number of genes per species which are conserved in all four, or only a subset of species shown in (**A**). The right-most pie chart shows numbers of conserved genes averaged across all three species*.*

We compared the identified sets of genes between all four dipteran species. For this purpose, we used transcriptome data from the modENCODE project for *D. melanogaster*[50]. As shown in Figure 3A, there is a large overlap between data sets, as a large number of genes is expressed in early embryos of all four species. Nevertheless, our analysis predicts a significant number of genes, which are specific to only a subset of species analyzed. The extent of overlap between data sets does not seem to correlate with phylogenetic distance (Figure 3B). Assuming that we are not missing a significant proportion of expressed genes, this indicates considerable plasticity in early development across different species, a phenomenon which has previously been described in drosophilid flies [9].

To further investigate the nature of this plasticity, we have carried out an enrichment analysis for gene ontology (GO) terms across species [57,58]. Detailed results from this analysis are shown in Additional file 3. They reveal that the range of GO categories is wide in all three species. Apart from a slight enrichment in transmembrane factors in *C. albipunctata* and *M. abdita*, we found no biologically significant differences between data sets. Furthermore, analysis of species-specific genes did not yield any obvious enrichment (data not shown). This is not surprising, since early embryogenesis is strongly conserved among dipterans (reviewed in [20,59,60]; most morphological differences described so far involve extra-embryonic tissues [61]). Therefore, similar spectra of gene functions are to be expected, while plasticity between species is most likely to involve temporal or spatial changes in gene expression, or different factors carrying out similar biological functions.

### Phylogenomics

To obtain evolutionary insights from our newly sequenced dipteran transcriptomes, we performed an exhaustive phylogenomic analysis in the context of sixteen other dipteran species with fully sequenced genomes (see Methods). This includes twelve *Drosophila* genomes [10,12,13], and four mosquitoes [11,14,15]. In addition, we included the lepidopteran *Bombyx mori*[62], and the coleopteran *Tribolium castaneum*[63] as outgroups. Our phylogenomic analysis consists of the reconstruction of a phylogenetic tree for every gene in the transcriptome. Such a set of gene trees is called a phylome [64]. This approach has been successfully applied to the analysis of genomes [65,66], but not yet to transcriptomes. Therefore, our transcriptomic data sets provide a unique opportunity to assess the performance of large-scale phylogenetic analyses on this type of data.

An expected limitation of reconstructed transcriptomes, as compared to whole genomes, is that reconstructed genes may be incompletely assembled. This is likely to affect the retrieval of homologs as well as subsequent steps in the phylogenetic reconstruction. Our pipeline successfully reconstructed phylogenetic trees for 77.5%, 71.2%, and 62.3% of the genes identified in the *C. albipunctata, M. abdita,* and *E. balteatus* transcriptomes, respectively. This is much smaller than the 91.1% coverage of the genome-based *D. melanogaster* phylome deposited in PhylomeDB [67]. However, these figures are hardly comparable: a transcriptome-based phylome will necessarily miss the genes not expressed at the relevant developmental stage. Furthermore, there are several closely related species for *D. melanogaster*, which facilitates the identification and retrieval of homologs.

Nevertheless, a comparison of coverage among the three transcriptome-based phylomes is informative, since they are based on similarly divergent species and represent similar developmental stages. In this context, the smaller coverage of the *E. balteatus* phylome is likely to indicate a lower quality and/or coverage of this transcriptome.

In support of this, we found that the number of homologs that could be retrieved by searching with BLAST with a given transcript as a query (i.e. homologs included in the tree) correlates significantly (Pearson correlation, *p*<<0.0001, in all three phylomes) with the length of the transcript sequence relative to the length of its *D. melanogaster* ortholog. In other words, more complete transcripts were able to detect a larger number of homologs.

In addition, the lower coverage observed for the *E. balteatus* phylome also seemed to result from a lower average number of homologs per gene tree (24.0) as compared to those in the *C. albipunctata* (34.1)*,* and *M. abdita* (33.5) phylomes. Taken together, this suggests that transcript length in the seed transcriptome determines coverage in terms of reconstructed trees and detected homologs in the resulting phylome. This, in turn, may result in errors during downstream analyses as shown before for low-coverage genomes [68].

The use of a reasonably closely related species with a complete genome (e.g. *D. melanogaster*) as an alternative seed could help to alleviate this problem, at least for those genes in the target species that have homologs in the alternative seed species. To test this, we reconstructed a new phylome comprising the same set of species but using the *D. melanogaster* genome as a seed. Our results show that trees reconstructed from *D. melanogaster* seed genes include a larger number of homologs (73.5), while still covering a significant part of the target transcriptomes (59.8% for *C. albipunctata,* 56% for *M. abdita,* and 35.1% for *E. balteatus*).

Finally, a combined phylome resulting from the addition of trees reconstructed from non-drosophilid species-specific transcriptome seeds whenever a transcript is not covered in the *D. melanogaster* phylome provides the highest coverage over the target transcriptomes (83.3% for *C. albipunctata,* 80.1% for *M. abdita,* and 65.8% for *E. balteatus*) while ensuring the maximal quality of each individual tree. We therefore adopted the combined phylomes for our subsequent analyses and recommend this as a general approach in future phylogenomic analyses of newly obtained transcriptomes.

Gene phylogenies can serve to accurately establish orthology and paralogy relationships across species [69,70]. We used an automated, phylogeny-based pipeline to produce a comprehensive catalog of orthologs and paralogs among the 17 insects considered, and annotated 1,514 (*C. albipunctata*), 1,690 (*M. abdita*) and 690 (*E. balteatus*) transcripts based on gene ontology terms transferred from functionally annotated orthologs, of which 1,279, 1,428, and 634, respectively were based on one-to-one orthology relationships. This catalogue and functional assessment will clarify equivalences among genes in different model organisms and facilitate future comparative analyses. All phylogenetic trees alignments and orthology and paralogy predictions are available through the PhylomeDB (http://phylomedb.org) and diptex (http://diptex.crg.es) databases (see Methods).

### Deep dipteran phylogeny

The deep phylogenetic relationships between basally branching dipteran lineages are not firmly established, particularly with respect to the position of the family Psychodidae, to which *C. albipunctata* belongs. Initial analyses based on molecular and morphological data suggested Psychodida and closely related families (Psychodomorpha) as the sister group of Brachycera, and this has been the predominant view (see [48], and references therein). However, recent analyses based on a combination of 18S and 28S ribosomal RNA genes, complete mitochondrial genomes, and up to 12 nuclear-encoded proteins, have tentatively placed Psychodomorpha as a sister group to Culicomorpha (mosquitoes and blackflies; cf. Figure 1A) [49].

**Figure 4:**
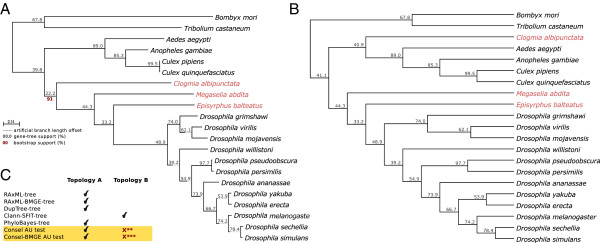
**Dipteran Phylogeny.** This figure shows two alternative topologies obtained from the phylogenetic analysis of 21 species, differing only on the position of *C. albipunctata*. Numbers above branches (in black) indicate the percentage of individual gene trees from the four reconstructed phylomes supporting each bipartition. (**A**) Hypothesis supported by most of the phylogenetic methods tested, including maximum likelihood analysis of 160 concatenated genes and one supertree approach (DupTree-tree). Branch lengths and bootstrap values (in red) correspond to the RAxML-tree (see Methods). Bootstrap values were calculated for all branch points. For clarity, we only show the one for the branch leading to *C. albipunctata*. (**B**) Alternative topology supported by one supertree approach (Clann-SFIT-tree). Branch information and bootstrap supports are not available with this methodology (**C**) Summary of all phylogenetic results: four methods supported topology A, while only one supported topology B. We also show results from CONSEL-based hypothesis testing (yellow background; Approximately Unbiased (AU) Test, see Methods). These results indicate significant *p*-values for the rejection of topology B both in the case of raw and BMGE-corrected alignment (*p* = 0.071 and 0.027 respectively).

Our transcriptomes of species from this and other basally branching lineages provide a unique opportunity to re-assess their phylogenetic relationships using an extended molecular data set. To do so, we selected 160 gene families that displayed strict one-to-one, phylogeny-based orthology relationships across all species considered. This constitutes thus far the largest phylogenetic data set to assess the debated position of basal dipterans. A Maximum Likelihood analysis of the concatenated 160-gene data set produced a highly-supported topology (RaxML-tree, Figure 4A).

We assessed the existence of compositional bias in our dataset using a principal component analysis of amino acid distributions (see Methods). Our results (Additional file 4: Figure S8) show that the three transcriptomes considered here have rather divergent amino acid compositions, different between each species and also different from other sequenced dipterans. To rule out a possible effect of the compositional bias in the obtained topology, we applied a trimming recoding method to minimize compositional heterogeneity, as implemented in BMGE [71]. The trimmed alignment produced exactly the same topology as shown in Figure 4A, using both Maximum Likelihood (RaxML-BMGE-tree) and Bayesian (PhyloBayes-tree) approaches. This topology is fully congruent with the established species relationships across mosquitoes [72] and *Drosophila* species [73], illustrating the ability of our data to recover known phylogenetic signal.

With respect to the position of *C. albipunctata*, our results are consistent with Psychodomorpha being the sister-group of Brachycera (including cyclorrhaphans such as *D. melanogaster, E. balteatus,* and *M. abdita*), and thus, is in contrast with the molecular study by Wiegmann et al. [49].

With respect to the branching order within Cyclorrhapha, on the other hand, our analysis is congruent with that of Wiegmann et al. [49]. It corroborates the fact that Syrphidae (*E. balteatus*) are more closely related to schizophoran flies (e.g. drosophilids) than Phoridae (*M. abdita*). This has important implications for the study of the evolution of developmental features such as the presence of the anterior morphogen Bicoid (Bcd) and the reduction of extra-embryonic tissues into a dorsal amnioserosa within the cyclorrhaphan lineage [28,30,46,47,49].

An alternative approach to reconstruct species relationships from multiple genes is the reconstruction of supertrees by combining the topological information of individual gene trees [4]. We implemented this by using two alternative parsimony approaches, one that finds the topology, which results in the least number of duplications when all the individual gene trees are reconciled, as implemented in DupTree [74], and one that renders the topology which is most congruent with all the gene trees in terms of observed bipartitions (SFIT), as implemented in Clann [75]. While the first supertree approach resulted in a topology that was fully congruent with that in Figure 4A, the second one rendered a slightly different topology (Figure 4B): here, *C. albipunctata* appears as sister group to mosquitos, consistent with Wiegmann et. al. [49]. This latter result reflects a larger gene tree support (average congruence with individual gene trees) in the relevant node for the scenario in Figure 4B (41%) as compared to that in Figure 4A (22%).

Thus, our two independent supertree approaches provide conflicting results with respect to the position of *C. albipunctata*, which correspond to (a) the classical scenario in which *C. albipunctata* is a sister group of Brachycera [48], and (b) the most recently supported topology by Wiegmann et. al. [49] in which *C. albipunctata* is the sister group of Culicomorpha. To compare both scenarios, we reverted to topological testing using a Maximum Likelihood framework and the Approximately Unbiased (AU) test [76], as implemented in CONSEL [77]. Both topologies shown in Figure 4 were tested, allowing for free optimization of the branch lengths, and computing their likelihood on the alignment of 160 orthologous genes, both before and after correcting for compositional heterogeneity.

Consistent with our results above, the clustering of *C. albipunctata* with Brachycera received stronger statistical support in both cases. Notably, the second scenario, in which *C. albipunctata* is the sister branch of Culicomorpha, could only be discarded (p<0.05) after compositional heterogeneity correction. This suggests that the compositional heterogeneity present in the data disrupts the main signal observed in the alignment in favor of the second topology.

Phylogenetic artifacts such as long-branch attraction or compositional bias are known to have a stronger effect in individual phylogenies, where the number of informative residues is smaller [4]. Thus, methods like gene concatenation, which directly—rather than indirectly, as in supertree approaches—use the combined information of gene sequences are generally considered more robust [4]. The sparse taxonomic sampling of basal dipterans for which genomic data is available results in relatively long branches for the three groups involved in the conflicting relationships (*C. albipunctata*, Brachycera, and Culicomorpha). This, together with the fact that transcriptomic data are incomplete, makes our individual gene tree dataset prone to errors, particularly with respect to the position of the three species where only transcriptomic data is available.

Note that the gene tree parsimony approach used by DupTree is expected to be robust to missing data (e.g. from incomplete transcriptomic data), whereas the split fit approach used by Clann is more sensitive [74,78]. Finally, our results point to the presence of compositional heterogeneity in the data, which favors the branching of *C. albipunctata* with mosquitoes. Taking all this into consideration, the results based on the concatenation of 160 conserved genes with additional support from one of the supertree approaches, provides strong support for the placement of *C. albipunctata* as the sister group of the Brachycera.

### Gene duplications and gene family expansion

**Figure 5:**
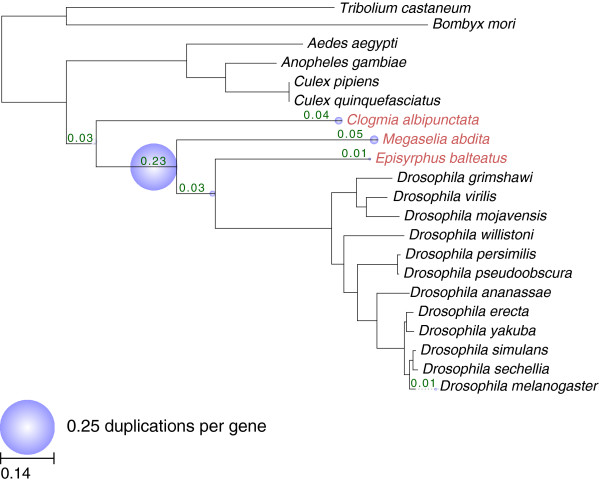
**Rate of duplications per gene in dipteran lineages.** Numbers above branches correspond to computed duplication rates (per gene) in the corresponding lineage. Superimposed bubbles are proportional to these numbers. All computations are based on the *D. melanogaster* phylome, except those specific to *C. albipunctata*, *M. abdita*, and *E. balteatus,* which are based on their corresponding phylomes (see main text for details).

Gene duplication is considered one of the major sources for functional innovation [79]. Analyses of complete eukaryotic genome sequences have revealed that gene duplication has been rampant, and that this process can be linked to important evolutionary transitions or major leaps in development and adaptive radiations of species (see, for example, [80,81]). To reconstruct the history of duplications for the genes identified in our transcriptomes within the dipteran lineages considered here, we used a phylogeny-based method to detect and date gene duplication events [69,82], and calculated the average number of observed duplications per gene in each of several relevant lineages in our phylogeny (Figure 5).

On average, 38% of the genes analyzed have experienced at least one duplication event in any of the lineages studied. The distribution of duplications across lineages shows a somewhat larger duplication rate in the cyclorrhaphan lineage (see large bubble in Figure 5), which may reflect a larger evolutionary distance represented by this branch. Duplications specific to each particular lineage were generally low, affecting less than 5% of the genome. Of note, roughly 3,000 duplications occurred during the period extending from the separation of mosquitoes from other dipterans up to the separation of the aschizan (*E. balteatus*) and schizophoran (*D. melanogaster*) lineages. This shows the utility of our newly generated transcriptomes for providing a more accurate picture of the evolutionary period at which the different gene families were duplicated.

## Conclusions

In this paper, we have presented a comparative transcriptomic analysis of three non-drosophilid dipteran species: *Clogmia albipunctata, Megaselia abdita,* and *Episyrphus balteatus*. These species are located at informative positions within the dipteran phylogeny, and constitute emerging model systems for comparative embryology and physiology. Our results indicate a high degree of conservation in gene expression during early development in dipteran insects. They are important both from a methodological, and a phylogenetic point of view.

In terms of methodology, we show that high-quality *de novo* assembly of transcriptomes can be achieved using Illumina sequencing technology with the Trinity assembly pipeline. The resulting transcriptomes are not only useful as resources for gene cloning and expression analysis, they also enable comparative and phylogenomic investigations that are more systematic and robust than those based on ESTs or selected candidate genes. 454 sequences (assembled by Newbler) are only required if accurate predictions of alternative splicing events are needed. With respect to phlyogenomic analyses, we obtained the most comprehensive sets of gene trees when combining phylomes in the following manner: first, we used a sufficiently closely related seed species with a sequenced genome (*D. melanogaster*), and then combined the trees derived from it with additional ones that are only present in phylomes based on the transcriptomes of each non-model species.

Our most important result, however, re-opens the discussion about deep dipteran relationships, which are difficult to resolve due to a rapid early radiation of flies, midges, and mosquitoes. A recent study, based on a large sample of species but a restricted amount of sequences from a selected subset of genes, placed psychodid midges such as *C. albipunctata* with the culicomorph branch of the Diptera, which includes the mosquitoes and blackflies [49]. In contrast, our phylogenomic analysis, based on a much larger sample of genes, suggests that the psychodids are a sister group of the brachycera, or ‘higher flies’, which includes phorids (*M. abdita*), syrphids (*E. balteatus*), as well as the drosophilids. This is consistent with the placement of the psychodids in earlier phylogenetic analyses (see, for example, [48], and references therein).

In addition to trees based on concatenated sequences, our analysis included the use of so called supertree approaches, which combine the information obtained for thousands of individual gene trees. In this case, the use of alternative optimization criteria provided ambiguous support for the clustering of *C. albipunctata* with either Brachycera or Culicomorpha. Our analysis indicates that this ambiguity is due to the presence of compositional bias, which favors the clustering of *C. albipunctata* with Culicomorpha. It seems that individual gene trees (many of which are based on incomplete transcriptomic data) are more strongly affected by compositional bias resulting in pervasive presence of the alternative signal. This is further corroborated by the fact that we can overcome this problem through the concatenation of a sufficient number of the most completely sampled genes, and by application of methods to correct for compositional heterogeneity. Both of these measures result in strong support for the classical affiliation of *C. albipunctata* as sister group of Brachycera.

All of the evidence described above points towards a grouping of Psychodidae with Brachycera. However, it remains controversial whether high species sampling or high sequence coverage yields more reliable phylogenetic trees [83-87]. Therefore, we cannot yet conclusively determine the position of *C. albipunctata*. Future studies with both a larger number of species, and a higher sequence coverage will be required to resolve these deep evolutionary issues.

## Methods

### Genomic library preparation and sequence acquisition

Total RNA was collected from 0–4½-hour old *Megaselia abdita*, and 8-, 10- and 12-hour old *Clogmia albipunctata* embryos (all raised at 25°C) using Trizol. cDNA was synthesized using the SMART cDNA library construction kit from Clontech (cat. no. 634901), with the CDS-3M adapter from the Evrogen Trimmer cDNA normalization kit (cat. no. NK002). We used the SuperScript III (Invitrogen) enzyme for reverse transcription, and Advantage 2 polymerase (Clontech) for library amplification.

The Trimmer-Direct cDNA normalization kit (Evrogen) was used to normalize and further amplify the cDNA library. Briefly, 100 ng of purified cDNA were incubated at 95°C for 2 min followed by incubation at 68°C for 5 h in the hybridization buffer included in the kit (50 mM Hepes, pH7.5, and 0.5 M NaCl). After the incubation, the reaction was treated with 0.25 units of duplex specific nuclease (DSN). The normalized cDNA was then amplified from 1 μl of DSN-treated cDNA in an 11-cycle PCR reaction using Phusion High-Fidelity DNA polymerase (New England Biolabs). The resulting amplified material was used for the preparation of normalized libraries (454 or Illumina) as described below.

454 library construction was performed as described in the GS FLX Titanium General Library Preparation Method Manual (Roche) with slight modifications. Briefly, 1.5 μg of the final normalized cDNA population was sheared to a size of 500 bp using the Covaris system, or by enzymatic fragmentation by incubation for 3 min at 37°C with 1.4 μl of dsDNA fragmentase (New England Biolabs) in a reaction volume of 14 μl. The fragment ends were made blunt and adaptors, which provide the priming sequences for both amplification and sequencing of the fragments, were ligated to both ends. These adaptors also provide a sequencing key (a short sequence of four nucleotides), which was used by the system software to recognize legitimate library reads. Next, the library was immobilized onto streptavidin beads, facilitated by a 5' biotin tag on Adaptor B. Finally, the unbound strand of each fragment (with 5'-Adaptor A) was released, and the quality of the recovered single-stranded DNA library was assessed using the Agilent 2100 Bioanalyzer (Agilent Technologies). Thereafter, the samples were quantified by qPCR using a KAPA library quantification kit (KAPA Biosystems), followed by emulsion PCR titration, large-scale emulsion PCR and sequencing on the 454-FLX sequencer using Titanium chemistry.

Illumina sequencing libraries were prepared from normalized, fragmented cDNA (same input as for 454 library preparation), by ligation to Illumina paired-end adapters following end-repair and A-tailing. Illumina libraries were quality-confirmed on the Bioanalyzer and, following KAPA quantification, were sequenced on the Illumina HiSeq 2000 using HiSeq v1 flow cells and sequencing chemistry.

Note that none of the sequencing protocols described above are strand-specific.

*Episyrphus balteatus* 454 reads (3–6hr zygotic data set from [42]) were downloaded from the NCBI Short Read Archive (SRA: http://www.ncbi.nlm.nih.gov/sra; id: SRR190625).

Tagdust [88] was used to eliminate reads containing homology to Illumina reads and to the cDNA adapter from the data prior to assembly with Trinity. Reads from the 454 platform were assembled separately by using Newbler v2.5.3 (Roche Diagnostics) with its -cdna option, as well as in combination with Illumina reads by using Trinity [7] on a server with 256 GB of RAM. Trinity was run with ‘--min_contig_length=100’ and ‘--bfly_opts --edge-thr=0.16’ options. Size distribution graphs were produced using R (http://www.r-project.org).

### Sequence assembly and functional annotation

Assembled sequences were annotated in two ways. For comparison of assemblers (Newbler versus Trinity), sequencing approaches (454 versus Illumina), and comparative analyses between species, we used BLASTx [51] against *Drosophila melanogaster* proteins (Ensembl Version 58, corresponding to FlyBase release 5.13) using an *e*-value limit of 10^-6^. Only the best hit was considered for annotation.

For phylogenomics and the finalized data sets in our database (see below), we re-annotated transcriptome sequences as follows: identified transcripts were translated in all six possible open reading frames (ORFs). For each detected ORF, a custom-made processing pipeline identifies protein signatures, assigns best orthologs, and uses orthology-derived information to annotate metabolic pathways, multi-enzymatic complexes, and reactions. First, ORFs are inspected for the presence of different protein signatures (such as families, regions, domains, repeats, and sites) by using InterProScan [89] and the InterPro database [90]. These signatures are used for the classification and automatic annotation of protein sequences by assigning biological functions and gene ontology (GO) terms. Second, each ORF is mapped to the UniRef50 protein database (http://www.ebi.ac.uk/uniref; [91]) using the BLASTp algorithm [51] in order to assess similarity with known protein sequences from other species. Finally, best-hit protein identifiers are then used to retrieve metabolic pathways, multi-enzymatic complexes, and reaction information available in the Reactome database (http://www.reactome.org; [92]).

Annotations obtained in this way were stored in a relational database based on MySQL (http://www.mysql.com). A public interface is available online at http://diptex.crg.es. Raw sequence reads for *M. abdita* and *C. albipunctata*, are available at the European Nucleotide Archive (ENA), accession number: ERP001635 (http://www.ebi.ac.uk/ena/data/view/ERP001635).

Proportional Venn diagrams for assembler and sequencing comparison as well as cross-species comparisons (Additional file 1: Figure S6) were created using the http://www.venndiagramk.tk web-tool by Tim Hulsen.

### Whole-mount *in situ* hybridization

Primers were designed from transcriptome sequences and amplified by PCR. Fragments were cloned into the PCRII-TOPO vector (Invitrogen) and used to make DIG-labeled riboprobes. Early, wild-type embryos of *M. abdita* and *C. albipunctata* were collected as described in [93] and [38]. Embryos were heat-fixed using a protocol adapted from [94], and were stained using a shortened version of the protocols of Tautz et al. [95] and Kosman [96], which is described in detail in [97]. For *C. albipunctata* embryos, the following modifications to the staining protocol apply: *proteinase K treatment* was carried out for 7 min at room temperature; *post-hybridization washes:* an additional wash of 10 min 2xSSC/hybridization buffer was performed before washing for 15 min with PBT/hybridization buffer; *antibody incubation:* embryos were incubated with anti-DIG for 2 hrs.

### Verification of alternative transcripts

We selected isogroups containing two alternative transcript variants as predicted by the assemblers (see Results). Two data sets were used: transcriptomes obtained with the 454 platform and assembled by Newbler v2.5.3 (Roche Diagnostics), and the combination of 454 reads with Illumina reads assembled by Trinity [7]. Ten pairs of primers were designed for each species and for each data set (40 in total) to detect the two predicted transcript variants of each gene. cDNA of the same stage as the transcriptome was used to amplify the putative splice variants by PCR, using different experimental conditions according to the primer pair (see Table S6 in Additional file 2). The size of PCR products was assessed by electrophoresis using agarose gels of different concentrations.

### Phylome reconstruction

The complete collection of gene phylogenies (known as the phylome) was reconstructed for *D. melanogaster,* as well as for *M. abdita, C. albipunctata*, and *E. balteatus.* The same taxon sampling was used for all phylomes, including 17 fully sequenced genomes (*Caenorhabditis elegans, Daphnia pulex, Ixodes scapularis, Acyrthosiphon pisum, Tribolium castaneum, Bombyx mori, Aedes aegypti, Anopheles gambiae, Culex pipiens, Culex quinquefasciatus, Drosophila melanogaster, Drosophila mojavensis, Nasonia vitripennis, Apis mellifera, Ciona intestinalis, Homo sapiens, Drosophila pseudoobscura, Pediculus humanus corporis*), and the three transcriptomes (*M. abdita*, *C. albipunctata*, and *E. balteatus*) analysed in this study. The automated phylogenetic pipeline described in [67] was used with the following modifications for the reconstruction of each phylome.

#### Homolog search

For each protein encoded in the *D. melanogaster* genome, a Smith-Waterman [98] search was performed against the rest of the 19 species (BLAST parameters: -FT –a 2 –s –z 1000000). Only significant hits (*e*-value <= 10^–5^) that aligned with a continuous region longer than 30% of the query sequence were selected (15% in the case of the *D. melanogaster* phylome). At most 200 sequences were taken for each query.

#### Alignment reconstruction

Multiple sequence alignments were built from each set of homologous sequences using M-COFFEE v8.80 [99] to combine the results of three different alignment programs: MUSCLE v3.8.31 [100], MAFFT v6.814b [101], and DIALIGN-TX [102]. Alignments were performed in forward and reverse direction, thus evaluating six alignments per query. The resulting alignment of each family was trimmed using trimAl v1.3 [103] using a consistency cutoff of 0.1667 and a gap score cutoff of 0.1.

#### Phylogenetic inference

For each set of homologous sequences, evolutionary model tests were performed prior to phylogenetic inference. For this, phylogenetic trees were reconstructed using a neighbor-joining approach as implemented in PhyML [104]. The likelihood of this topology was computed allowing branch-length optimization and using six different evolutionary models (JTT, WAG, MtREV, LG, Blosum62, DCMut), as implemented in PhyML 3.0 [104]. The model best fitting the data was determined by comparing the likelihood of all models according to the AIC criterion. A maximum likelihood tree was inferred using the best-fitting model. In all cases, a discrete gamma-distribution model with four rate categories plus invariant positions was used. The gamma parameter and the fraction of invariant positions were estimated from the data.

The resulting phylomes were uploaded to the PhylomeDB database [67], with the following internal identifiers: 174, 183, 184, and 191. Individual trees and alignments can be searched and downloaded from http://phylomedb.org.

### Combined phylome dataset

In order to maximize the coverage of our phylogenomic analysis, we generated a combined set of gene trees using the four reconstructed phylomes. The *D. melanogaster* phylome was used as the main source of trees, using only trees from the other three phylomes when a sequence from any of the transcriptomes from *C. albipunctata, M. abdita*, and *E. balteatus* was not represented in the *D. melanogaster* phylome. The combined set of trees (provided in full in Additional file 5) includes 16,894 gene-trees.

### Orthology-based functional annotation

For each *M. abdita* and *C. albipunctata* gene, Gene Ontology [105] annotations were transferred from its orthologs in *D. melanogaste*r. A species-overlap approach, described in [69], was used to scan the whole set of gene family trees obtained from the *D. melanogaster* phylome, and to discriminate all orthology relationships between genes from *D. melanogaster* and the three non-drosophilid species*.* The type of orthology (one-to-one, one-to-many, and many-to-many) was also discriminated for each prediction (data included in PhylomeDB; http://phylomedb.org).

### Detection of lineage-specific gene duplications

Lineage-specific gene duplications were inferred by analyzing all gene family trees in the *D. melanogaster* phylome with a previously described topology-based algorithm to detect and date duplication events [69,82]. Species-specific family expansions constitute a special case of duplications, in which paralogs of a single species are present. The fact that phylome data may report redundant information about the same evolutionary event (each homologous gene has its own tree) was taken into account, and redundant data were merged. Tree analysis was performed by using the methods provided in the ETE toolkit [106].

### Supermatrix tree reconstruction (RAxML-Tree)

We built a concatenated alignment based on 160 single-copy orthologous genes present in the complete set of 21 species considered. Trimmed alignments obtained from the phylome reconstruction pipeline were used for the concatenation phase. The final supermatrix contains a total of 55,303 columns partitioned in four blocks, each matching a different evolutionary model (DCMut, JTT, LG, WAG). Phylogenetic tree inference was performed using RAxML 7.2.8 [107] under the rapid hill-climbing algorithm (“-f d” option), using partitioned models. One thousand bootstrap replicates were calculated to provide branch supports.

### Calculation of compositional bias and corrected supermatrix tree (RAxML-BMGE-Tree)

Heterogeneity in amino acid composition among the sequences contained in the concatenated alignment used for the RAxML-tree was detected through a Principal Component Analysis (PCA), using a per-species vector of amino acid frequencies (see Additional file 4: Figure S8). The BMGE tool [71] was used to correct for compositional heterogeneity by trimming the concatenated alignment of the 160 single-copy orthologous genes used for in the RAxML-tree. A new ML tree (RAxML-BMGE-tree) was inferred based on the BMGE-corrected alignment using RAxML and the rapid hill-climbing algorithm (“-f d” option) as above.

### Supertree reconstruction (DupTree-Tree/Clann-SFIT-Tree)

The complete collection of 16,894 gene-trees (provided in Additional file 5) that resulted from the combination of the four generated phylomes (see above) were used to infer several supertree-based phylogenies. First, the TreeKO algorithm [108], and the ETE toolkit [106] were used to construct a list of 32,437 species-tree topologies represented in all gene evolutionary histories (provided in Additional file 6). This methodology decomposes multi-gene family trees into all implied subtrees containing only orthologs and speciation events, thus enabling the use of supertree methods that do not accept multi-labeled trees as an input (see [108], for details). To avoid redundancy, only speciation histories containing the seed sequence were kept. This final set of trees was used for all supertree approaches described below.

The DupTree tool [74] with default parameters was used to infer a species supertree (DupTree-tree). This program uses a gene-tree parsimony approach to find the species topology, which involves the least number of duplication events when a collection of gene trees is reconciled. In addition, another supertree method implemented in Clann [75], using Maximum Splits Fit (SFIT), was used (Clann-SFIT-tree). This approach finds the species topology that is most compatible in terms of tree bipartitions (splits) with a given collection of gene trees.

### Bayesian tree reconstruction (PhyloBayes-Tree)

Our previously generated BMGE-concatenated alignment (see RAxML-BMGE-Tree above) was used to perform a Bayesian phylogenetic reconstruction using PhyloBayes [109]. The analysis was executed using two independent Monte Carlo Markov Chains and the CAT model. Both chains converged into the same tree topology (maxdiff = 0, min. effective size = 56) using a burnin parameter of 1,000 trees.

### Calculation of gene tree support

Gene tree support values for all the branches in the species phylogenies were calculated as the percentage of individual gene trees within the combined set of 16,894 phylome trees supporting each bipartition.

### Topology hypothesis testing

The relative position of *C. albipunctata* was the only difference found among the topologies obtained from all phylogenetic analyses. The software CONSEL [77] was used in order to calculate the statistical confidence of the two alternative trees. For this, we proceeded as follows: (1) We created two artificial topologies in which all nodes remained unresolved except for the one defining the conflicting position of *C. albipunctata*. (2) Each of the constrained topologies was used to reconstruct a new maximum likelihood tree using the concatenated BMGE alignment and RAxML (“-f d” options). (3) Individual likelihood values for each column in the alignment were dumped (“-f g” RAxML option). (4) Per-site maximum likelihood values of both alternative topologies were tested using the Approximately Unbiased (AU) Test as implemented in CONSEL v0.20. The same analysis was repeated using the uncorrected RAxML-tree source alignment (see RAxML-Tree above).

## Competing interests

The authors declare that they have no competing interests.

## Authors’ contributions

EJG: performed experiments and contributed to data analysis and validation, JHC: performed phylogenomic and gene family analyses, LC: assembled, annotated, and analyzed transcriptome sequences, KRW: contributed experimental work, HK: developed sequencing protocols for the 454 Titanium platform, and prepared libraries for sequencing with 454 and Illumina technologies, HH: supervised sequencing work and primary data quality control, GR: supervised bioinfomatic work, contributed to assembly and annotation of transcriptome sequences, and set up the diptex database, TG: supervised and performed phylogenomic and gene family analyses, JJ: initiated and supervised the project, and contributed to data analysis. EJG, TG, and JJ wrote the paper (with additional contributions from the other authors). All authors read and approved the final manuscript.

## Supplementary Material

Additional file 1 **Transcriptome sequencing, assembly, and annotation.** Describes sequence data sets, *de novo* assembly, and automatic annotation in detail. Analyses are summarized in **Tables S1–S3**. Contains supplementary **Figures S1–5**, which show length distribution plots for 454 raw reads, contigs, and isotigs, and well as Trinity transcripts (contigs) for all assemblies and species. **Figure S6** shows a comparative analysis of annotations between species and assembly strategies. (PDF 535 kb) Click here for file

Additional file 2 **Verification of annotation.** Describes details of manual verification of transcriptome annotation not shown in the main text. Includes an analysis of predicted alternative splicing events. Contains supplementary **Tables S4 and S5** summarizing manual curation and presenting a detailed list of manually curated candidate genes. **Figure S7** and **Table S6** show details of the verification of alternative splice isoforms as predicted by Newbler- and Trinity-based assemblies. (PDF 4599 kb) Click here for file

Additional file 3 **GO term enrichment analysis.** Describes details of the enrichment analysis for GO terms associated with sets of genes present in different species, and for genes specific to subsets of species. Contains supplementary **Table S7**, with details of enriched GO categories between species. (XLSX 35 kb) Click here for file

Additional file 4 **Principal component analysis (PCA) of compositional bias.** Contains **Figure S8** showing the results of a PCA for amino acid distributions from concatenated sequences in all 21 species considered in our phylogenomic analysis. (PDF 112 kb) Click here for file

Additional file 5 **List of gene-tree phylogenies.** Text file containing all gene trees obtained from the combination of the four phylomes reconstructed in this study. (7Z 10204 kb) Click here for file

Additional file 6 **List of species-trees topologies.** Text file containing all topologies which are represented in the combined set of gene-tree phylogenies. (GZ 284 kb) Click here for file
