# Supplementary material for: Comparative transcriptomics of early dipteran development
Source: BMC Genomics. 2013 Feb 24;14:123. doi: 10.1186/1471-2164-14-123 (PMC3616871; doi:10.1186/1471-2164-14-123)
Supplement: Additional file 1 — Transcriptome sequencing, assembly, and annotation. Describes sequence data sets, de novo assembly, and automatic annotation in detail. Analyses are summarized in Tables S1–S3. Contains supplementary Figures S1–5, which show length distribution plots for 454 raw reads, contigs, and isotigs, and well as Trinity transcripts (contigs) for all assemblies and species. Figure S6 shows a comparative analysis of annotations between species and assembly strategies. (PDF 535 kb) [file 1471-2164-14-123-S1.pdf]

Additional File 1:
Transcriptome Sequencing, Assembly and Annotation

Section S1.1: Sequencing

Table S1. Sequence data sets. Comparison of the total amount of sequence, number of reads, and average read length for 454 pyro-sequencing and Illumina HiSeq in all three species. Note that two 454 sequencing runs were performed for *C. albipunctata* compared to one for each of the other species. 454 read length distributions are shown in Figure S1.

	454			Illumina		
	Total Length (Mb)	Number of Reads	Average Read Size (bp)	Total Length (Mb)	Number of Reads	Read Size* (bp)
C. albipunctata	253	808,314	312.6	4,619	46,193,132	50
M. abdita	110	397,117	276.8	8,463	84,630,878	50
E. balteatus	115	311,906	368.8	-	-	-

* Per paired end.

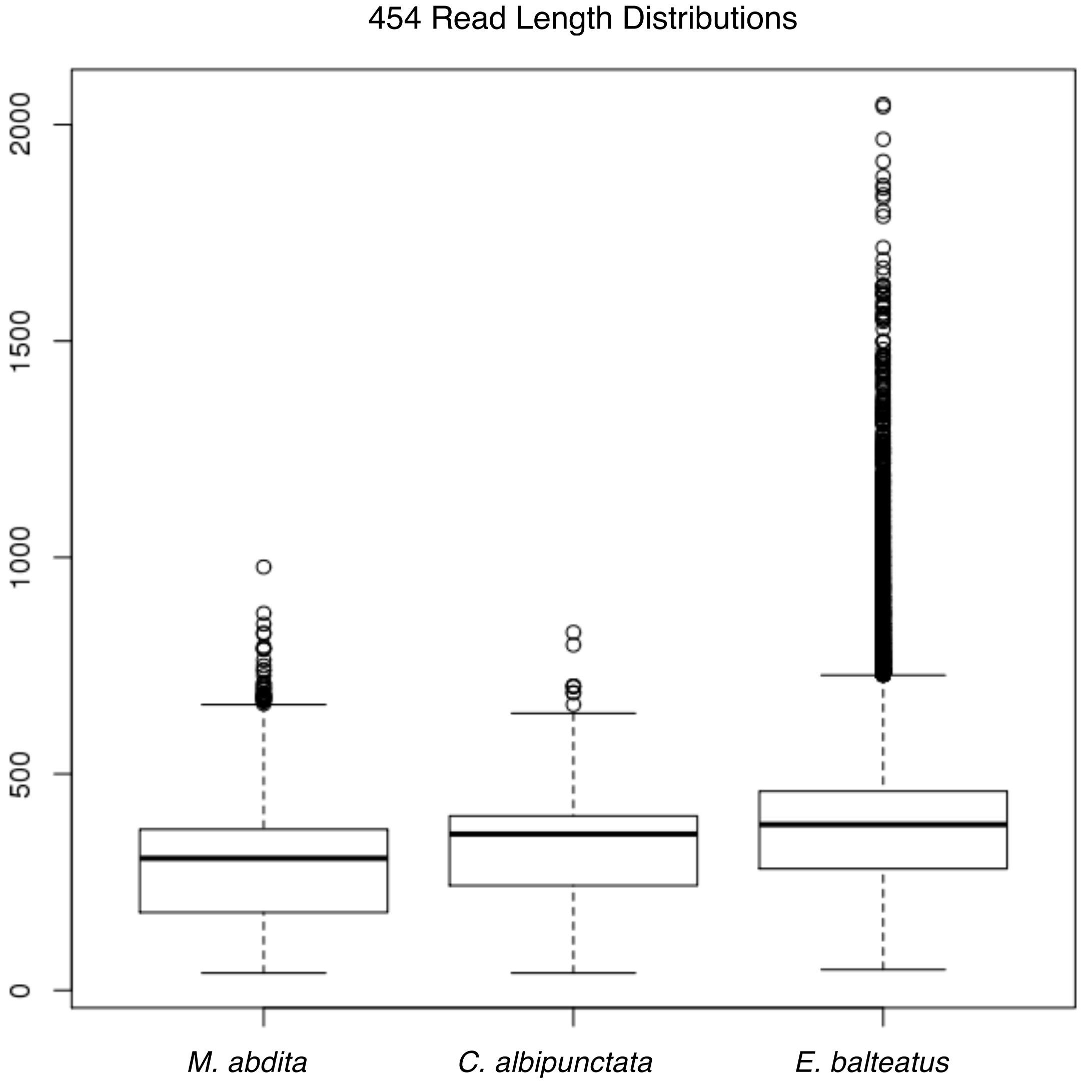

Figure S1: Box-and-whisker plots of length distributions for 454 raw reads per species. Thick black lines represent median read length. Extent of boxes represents quartiles. Extent of whiskers represents 1.5 inter-quartile range (IQR). Circles indicate outliers.

Section S1.2: Assembly

Newbler contigs roughly represent partial or full-length exons, which may be arranged into longer sequences — called isotigs — if several alternative arrangements of contigs are allowed. Isotigs represent transcripts consisting of several exons. They can be assigned to isogroups, which are sets of transcripts exhibiting sequence overlap. In this way, each isogroup can be thought of as representing a gene. Finally, reads that cannot be integrated into a contig are called singletons. Newbler assembled 69–82% of the reads into contigs (Table S2A). Table S2A also shows the number of contigs, isogroups, isotigs, and singletons, and the average/maximum length of contigs and isotigs in our 454 assemblies. Size distributions are shown in Figures S2 and S3.

Trinity is one of the few transcriptome assemblers currently able to analyze Illumina reads in the absence of a reference genome, and to identify both alternative transcripts and duplicated genes. It combines reads into unique transcript sequences (contigs) that can be grouped into contig clusters representing alternative transcripts of the same gene or closely related paralogs. In this sense, Trinity transcripts are comparable to isotigs in Newbler assemblies, and Trinity clusters to isogroups. We first tested Trinity on the 454-only data sets for comparison of assembler performance. 55–81% of reads were assembled into contigs (Table S2B). Size distributions are shown in Figure S4. The number of transcripts and clusters produced by Trinity on 454 data is higher than the one produced by Newbler, while the average transcript length is shorter. We then used Trinity to analyze our Illumina reads for *C. albipunctata* and *M. abdita*, both separately and in combination with 454 data. 54–58% of reads could be assembled into contigs (Table S2B). The assembly of Illumina-only reads shows a large number of clusters and transcripts with small average size. Adding 454 reads to Illumina data somewhat reduces the number of clusters, while slightly increasing the average transcript length (Table S2B; size distributions shown in Figure S5). However, both effects are small and did not lead to a marked improvement of the assembly, indicating that the Trinity assembler is unable to take advantage of such combined sequence data sets. This is expected, since Trinity does not take advantage of the increased read length from 454 sequencing.

Table S2. Sequence assembly. Numbers and average sizes for contigs, isotigs, and isogroups (A, Newbler assemblies), or transcripts and clusters (B, Trinity assemblies) for all three species. Asterisks in (B) indicate sequence datasets used in each assembly. Percentages of assembled reads were calculated against the number of raw reads before quality trimming. Size distributions for contigs, isotigs, and transcripts are shown in Figures S2–5.

A	Reads	Contigs			Isotigs			Isogroups	Singletons
	% of reads assembled	number	average length (bp)	max length (bp)	number	average length (bp)	max length (bp)		
C. albipunctata	81.9	13,520	950	9,038	12,209	1,211	9,038	10,151	37,574
M. abdita	68.8	11,245	521	4,028	10,205	655	4,028	8,599	63,762
E. balteatus	81.1	10,205	605	6,414	8,396	891	6,414	6,727	41,925

Table S2. (contd.)

B	Illumina	454		transcripts			clusters
			% of reads assembled	number	average length (bp)	max length (bp)	
C. albipunctata		*	81.0	25,160	960	9,032	14,561
M. abdita		*	54.7	16,694	519	3,484	12,807
E. balteatus		*	46.4	25,490	602	6,387	12,857
C. albipunctata	*		58.4	54,460	470	8,307	51,391
M. abdita	*		56.7	147,101	343	13,017	134,838
C. albipunctata	*	*	57.6	51,436	611	8,879	45,704
M. abdita	*	*	54.2	147,878	350	13,017	134,112

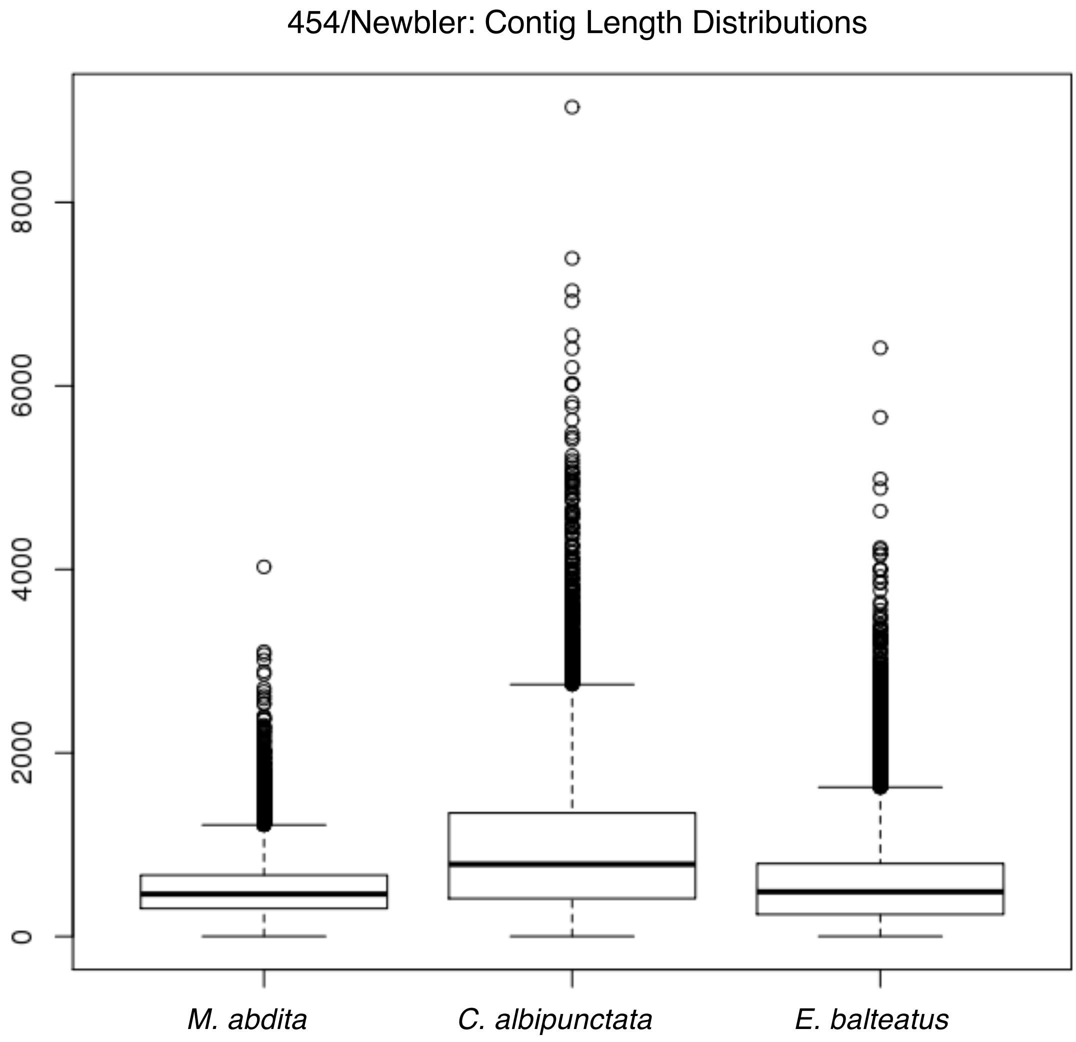

Figure S2: Box-and-whisker plots of length distributions for Newbler-assembled contigs (based on 454 sequencing) per species. Contigs roughly correspond to expressed exons. Thick black lines represent median read length. Extent of boxes represents quartiles. Extent of whiskers represents 1.5 inter-quartile range (IQR). Circles indicate outliers.

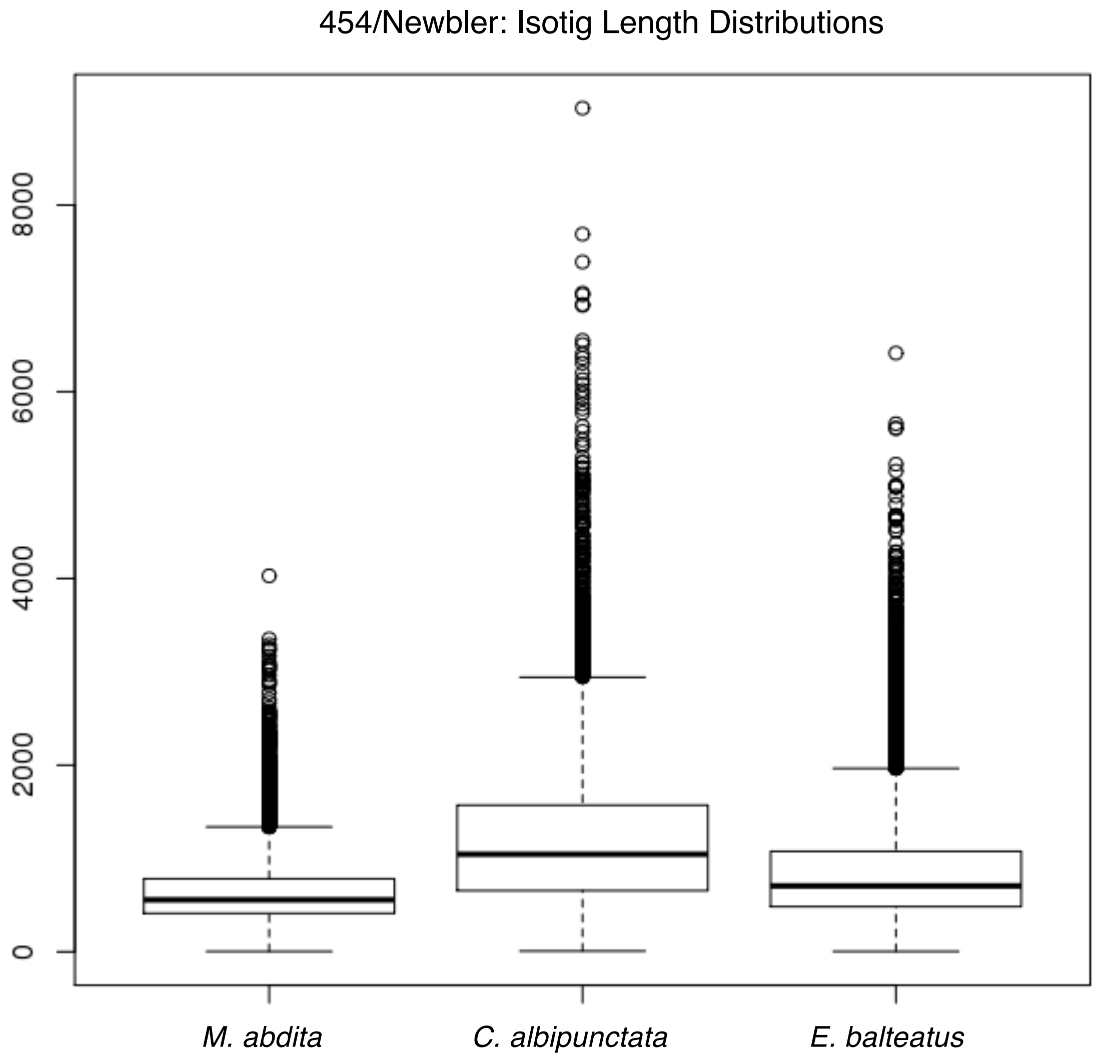

Figure S3: Box-and-whisker plots of length distributions for Newbler-assembled isotigs (based on 454 sequencing) per species. Isotigs roughly correspond to transcripts (cf. Figure S4). Thick black lines represent median read length. Extent of boxes represents quartiles. Extent of whiskers represents 1.5 inter-quartile range (IQR). Circles indicate outliers.

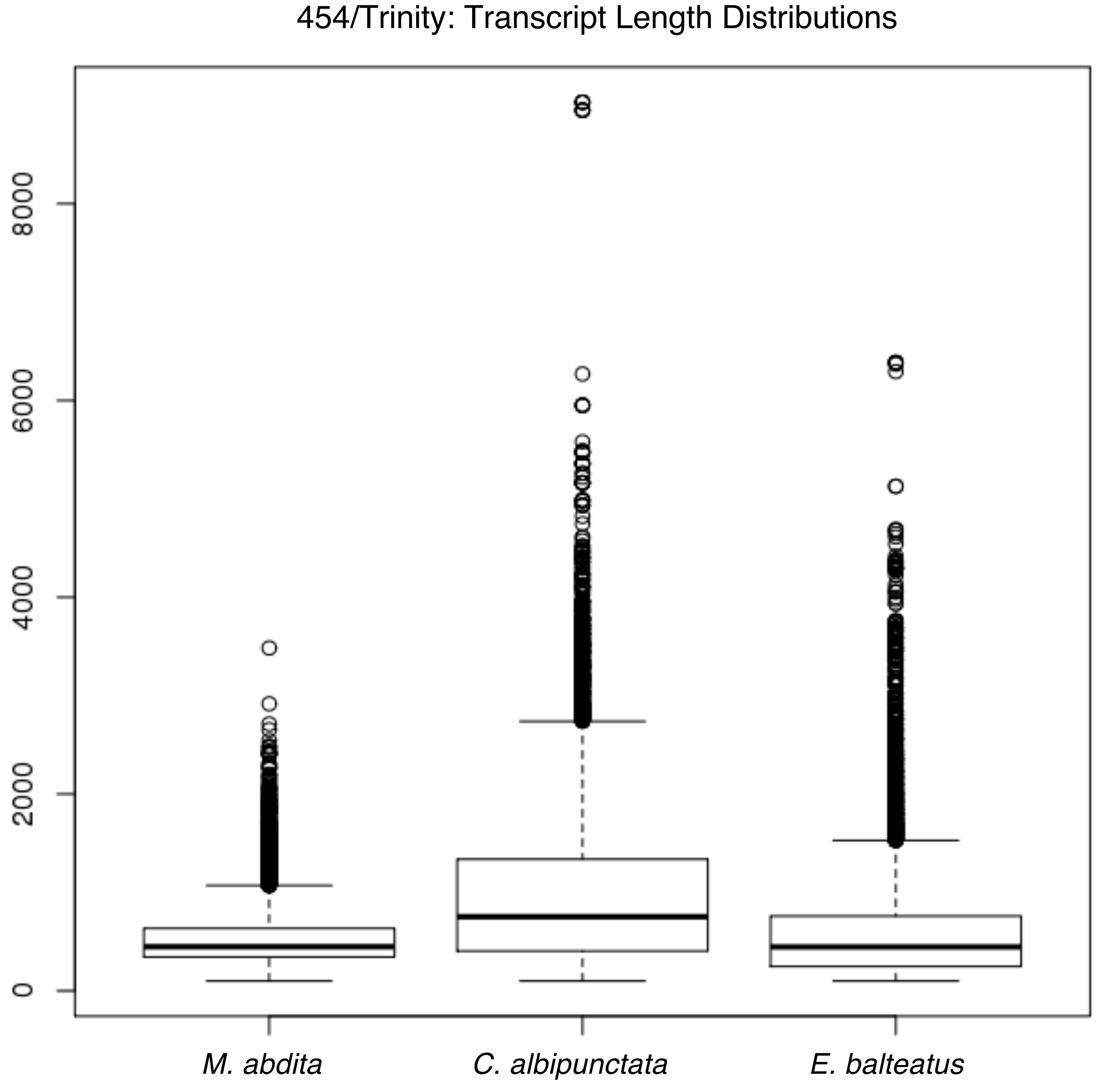

Figure S4: Box-and-whisker plots of length distributions for Trinity-assembled transcripts (based on 454 sequencing) per species. Transcripts are roughly equivalent to isotigs in Figure S3. Thick black lines represent median read length. Extent of boxes represents quartiles. Extent of whiskers represents 1.5 inter-quartile range (IQR). Circles indicate outliers.

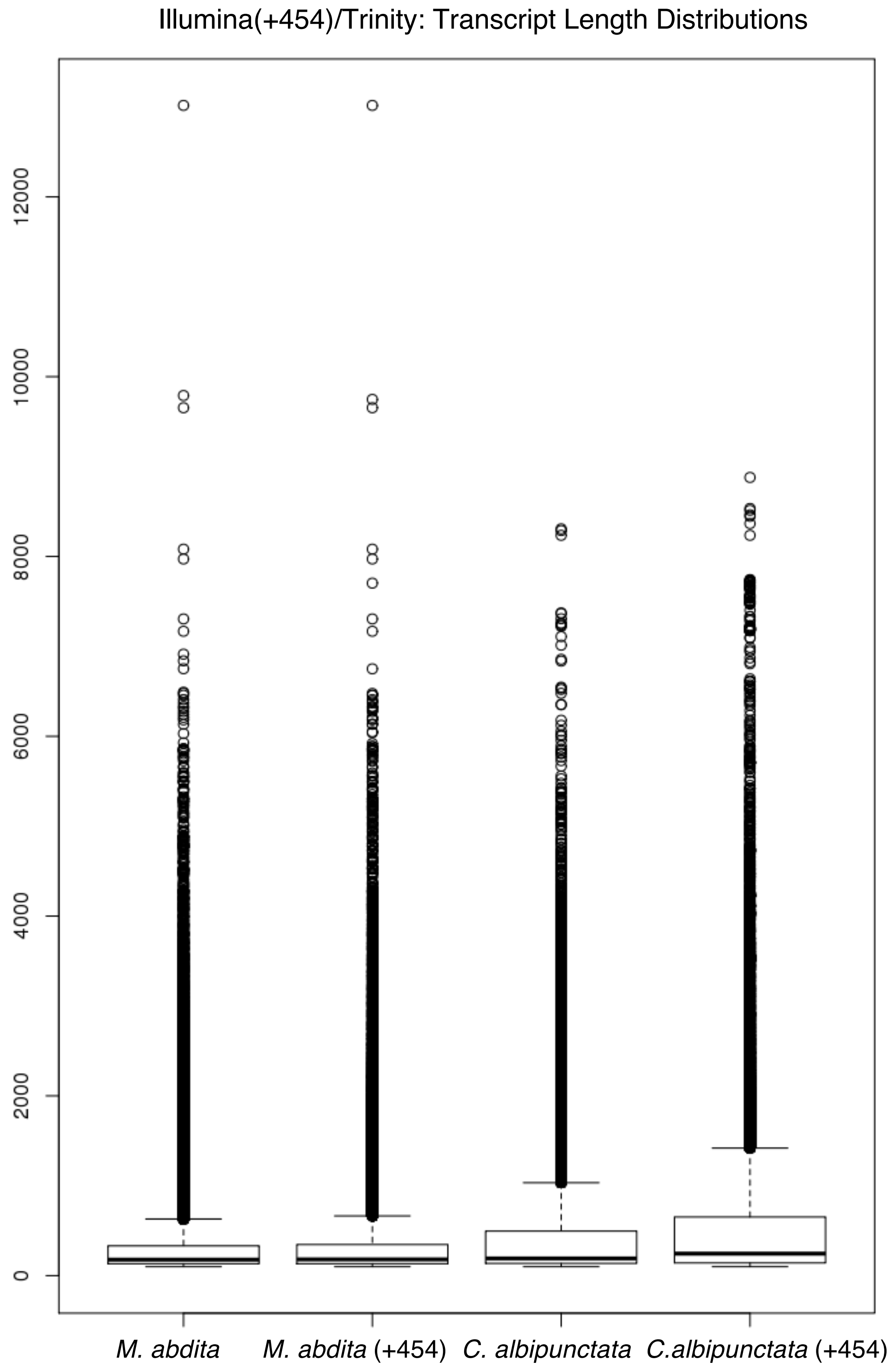

Figure S5: Box-and-whisker plots of length distributions for Trinity-assembled transcripts (based on Illumina with or without addition of 454 sequences) per species. Transcripts are roughly equivalent to isotigs in Figure S3. Thick black lines represent median read length. Extent of boxes represents quartiles. Extent of whiskers represents 1.5 inter-quartile range (IQR). Circles indicate outliers.

Section S1.3: Annotation

Table S3A shows annotation results for 454 reads assembled with Newbler. 63–77% of assembled isotigs have at least one BLAST hit, as opposed to only 20–27% for singletons. However, the number of *D. melanogaster* proteins matching singletons is considerable: for *C. albipunctata,* 4,680 proteins were represented in this way, and in *M. abdita* and *E. balteatus* the numbers of proteins matching singletons (7,528 and 6,584, respectively) are comparable to those matching isotigs (7,689 and 7,000; Table S3A). Genes with alternative transcripts can encode several proteins. Therefore, we also analyzed how many distinct genes are represented in our data sets. Out of 14,077 known and predicted *Drosophila* genes (Ensembl, Version 58) we detected 6,042–6,633 in each of the transcriptomes in our three species (Table S3A). Note that matches to singletons (but not isotigs) contribute between 854 and 2,092 genes to these numbers.

We also annotated assemblies based on 454 sequences with Trinity (Table S3B). These data sets generally show a lower number of matching *D.* melanogaster genes compared to the corresponding Newbler assemblies (compare Table S3A and B). Illumina data sets, on the other hand, show a dramatically increased number of BLAST hits, although only a low percentage of assembled contigs matches any *D. melanogaster* protein, and many hits turned out to be redundant. This results in a final number of matching genes which is only slightly higher than for Newbler assemblies based on 454 reads (Table S3A,B). Merging 454 and Illumina data did not significantly increase the number of hits or matching proteins/genes (Table S3B).

We compared the genes detected in 454-only data sets by both assembly methods. For every genome, singletons and isotigs identified in the Newbler assembly cover almost all the genes obtained when using Trinity, plus a significant number of additional genes that do not match any Trinity-assembled sequences (Figure S6A). In the case of Newbler, a vast majority of these additional genes are detected from the analysis of 454 singleton reads.

Next we compared the set of genes identified using Newbler (isotigs and singletons combined) with those obtained by assembling Illumina reads — separately and in combination with 454 reads — using Trinity (Figure S6B). The majority of genes can be found in all the assemblies. The number of genes uniquely identified by Newbler and 454 sequencing is quite small in both species (Figure S6B). Trinity and Illumina sequencing, on the other hand, predicts matches with a significant number of additional genes (Figure S6B). The closer match of uniquely predicted genes in *C. albipunctata* is likely a consequence of the higher coverage of 454 sequencing compared to *M. abdita.*

Our analysis concurs with an earlier study by Ewen-Campen et al. (2011; BMC Genomics 12: 61) showing that it is extremely important to include singleton reads in the analysis if only 454 sequences are available: while singleton fragments are less reliable and generally shorter than assembled sequences they still provide essential and useful information for the identification and cloning of a large number of genes.

Table S3. *Drosophila melanogaster* proteins and genes represented in our data sets. (A) shows the number and percentage of isotigs/singletons for Newbler assemblies, and (B) the number and percentage of transcripts for Trinity assemblies with BLAST hits against *D. melanogaster* proteins. The columns on the right show the number of unique proteins and gene sequences represented in each data set (isotigs and singletons together for Newbler assemblies). Starred numbers indicate genes matching only singletons but not isotigs. Note that genes with alternative transcripts can encode multiple proteins. *Clogmia* 454 data show a higher ratio of hits to isotigs versus singletons since two sequencing runs were done for this species. Asterisks in (B) indicate sequence datasets used for each assembly.

**A**		**# with BLAST hits**	**%**	**# of matching proteins**	**# of matching genes**		**total # of genes**
C. albipunctata	isotigs	8,218	67.3	9,309	5,779		6,633
	singletons	7,284	19.4	4,680	2,441	854*	
M. abdita	isotigs	6,399	62.7	7,689	4,539		6,457
	singletons	12,734	20.0	7,528	4,251	1,918*	
E. balteatus	isotigs	6,489	77.3	7,000	3,950		6,042
	singletons	11,391	27.2	6,584	3,847	2,092*	

**B**	**Illumina**	**454**	**# with BLAST hits**	**%**	**# of matching proteins**	**# of matching genes**
C. albipunctata		*	13,942	55.4	9,909	5,064
M. abdita		*	8,586	33.7	8,239	4,860
E. balteatus		*	14,582	57.2	8,852	6,075
C. albipunctata	*		14,776	27.1	11,217	6,660
M. abdita	*		16,612	11.3	12,612	7,721
C. albipunctata	*	*	13,514	26.3	11,110	6,686
M. abdita	*	*	16,618	11.2	12,623	7,741

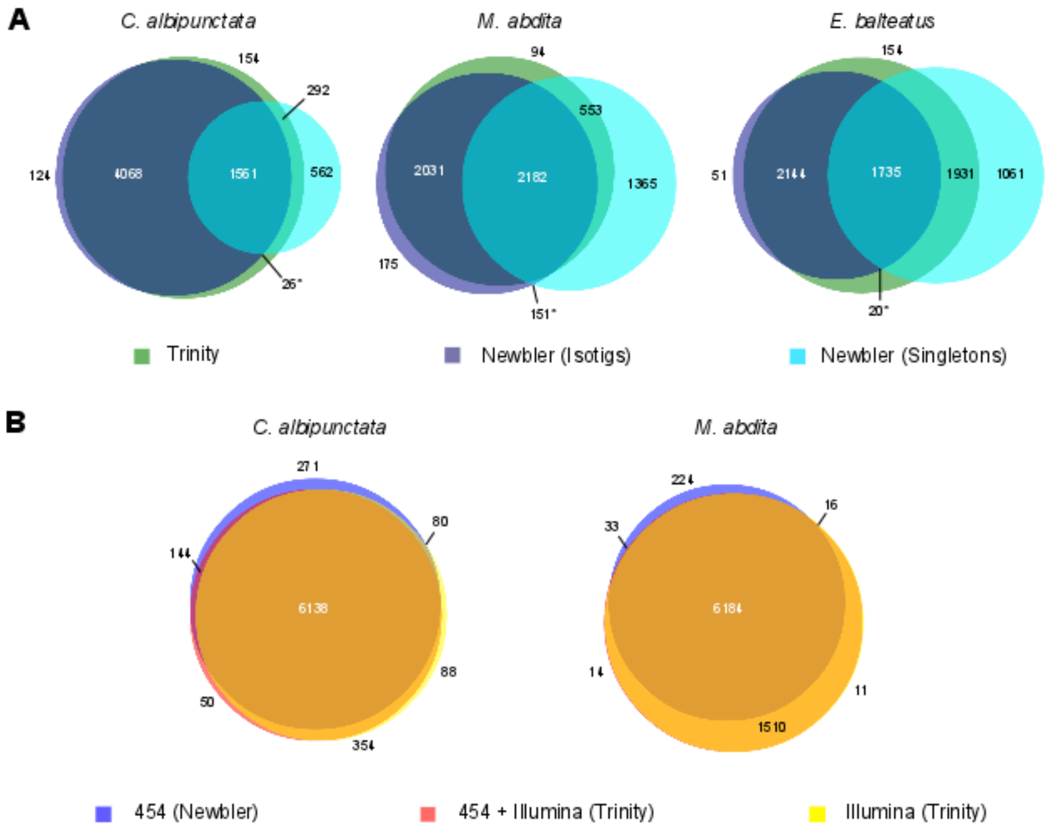

Figure S6: Comparison of transcriptome assemblies. (A) Proportional Venn diagrams comparing genes found in assemblies of 454 data. The assembly based on Trinity (green) is compared to Newbler isotigs and singletons (blue) for all three species. (B) Proportional Venn diagrams comparing genes found in 454 data assembled by Newbler (isotigs and singletons; blue) with the assembly of Illumina HiSeq data alone (Trinity; yellow), and a combined assembly of both 454 and Illumina HiSeq reads (Trinity; red). Illumina HiSeq data are only available for *C. albipunctata* and *M. abdita,* but not for *E. balteatus.* Asterisks mark areas that could not be drawn in correct proportion due to inherent geometrical constraints on Venn diagrams based on circles.
