# Supplementary material for: Comparative transcriptomics of early dipteran development
Source: BMC Genomics. 2013 Feb 24;14:123. doi: 10.1186/1471-2164-14-123 (PMC3616871; doi:10.1186/1471-2164-14-123)
Supplement: Additional file 2 — Verification of annotation. Describes details of manual verification of transcriptome annotation not shown in the main text. Includes an analysis of predicted alternative splicing events. Contains supplementary Tables S4 and S5 summarizing manual curation and presenting a detailed list of manually curated candidate genes. Figure S7 and Table S6 show details of the verification of alternative splice isoforms as predicted by Newbler- and Trinity-based assemblies. (PDF 4599 kb) [file 1471-2164-14-123-S2.pdf]

Additional File 2:
Verification of Annotation

Section S2.1: Verification by Manual Curation

We assessed the quality of our transcriptome annotation manually in the following way: we queried our transcriptome database using either search by gene name, or protein BLAST to check for the presence or absence of 107 candidate genes known to be expressed during the blastoderm stage and early germband extension in *Drosophila melanogaster.* We then used positive name searches and BLAST hits above a given expect (*e*-)value ($>10^{-5}$), and performed a reciprocal BLAST search of NCBI GenBank to test, whether the sequences correspond to true homologs, or mis-annotated paralogs in other species. The results of this analysis are summarized in Table S4. A detailed list of curated candidate genes is provided in Table S5.

Between 39% and 65% of the candidate genes can be found by name search, while the number of genes detected by BLAST is much higher (67–85%). This discrepancy indicates issues with gene nomenclature, and/or missing annotations for a relatively large number of genes ('false negatives' in Table S4), which will have to be improved by manual curation. On the other hand, the high number of positive BLAST hits indicates good coverage of our data sets. Only between 4 and 9% of candidate genes could not be found by BLAST search. This includes genes whose expression is not conserved or heterochronically shifted to later stages in non-drosophilid species. Finally, a considerable number of genes (9–24%) could be detected in our transcriptomes, but could not be verified by reciprocal BLAST. These genes possibly represent cases of mis-annotated paralogs in our data set. Again, this is not unusual for an automatic annotation pipeline, and can only be improved by further manual curation.

Table S4. Verification of transcriptome annotation. We chose 107 candidate genes known to be expressed during blastoderm stage and early germband extension in *D. melanogaster,* and verified their presence or absence in our annotated transcriptomes in *C. albipunctata, M. abdita,* and *E. balteatus* (see Table S5). We searched our transcriptomes by gene name, and protein BLAST using *D. melanogaster* homologs of each candidate gene. Positive name searches, and BLAST hits with an *e*-value >10^{-5} were then reciprocally BLASTed against the NCBI GenBank database (using tBLASTx) to verify whether they represent true homologs. 'Positives' indicates genes present in our transcriptomes that are properly annotated; 'false negatives' indicates genes that are present, but not properly annotated; 'negatives' are genes that are not present in the database; 'false positives' indicates genes are present in the annotation, but cannot be verified by BLAST.

	Clogmia albipunctata		***Megaselia abdita***		***Episyrphus balteatus***	
	Search by Name	BLAST	Search by Name	BLAST	Search by Name	BLAST
Positives	39.2%	67.3%	64.5%	85%	47.7%	68.2%
False negatives	30.0%	0.9%	21.5%	1.9%	21.5%	0.9%
Negatives	30.8%	9.3%	13.1%	3.7%	29.9%	6.5%
False positives	0.9%	22.4%	0.9%	9.3%	0.9%	24.3%

Table S5: List of manually verified candidate genes.

	C. albipunctata		*M. abdita*		*E. balteatus*	
	Blast Search	Name Search	Blast Search	Name Search	Blast Search	Name Search
Notch	Y	Y	Y	Y	Y	Y
Delta	Y	Y	Y	Y	Y	Y
Serrate	X	N	X	N	X	N
hairy	X	N	Y	Y	Y	N
Enhancer of split	X	N	X	N	X	N
fringe	Y	Y	Y	N	N	N
dishevelled	Y	N	Y	N	Y	Y
frizzled	Y	Y	Y	Y	Y	Y
armadillo	Y	Y	Y	Y	Y	Y
pangolin	N	N	N	N	N	N
prickle	X	N	X	N	X	N
Van Gogh	Y	Y	Y	Y	Y	Y
shaggy	Y	N	Y	Y	Y	Y
hedgehog	Y	N	Y	Y	N	N
patched	Y	Y	Y	Y	Y	Y
smoothened	Y	Y	Y	Y	Y	Y
cousin of atonal	X	N	X	N	X	N
mirror	X	N	Y	N	Y	Y
araucan	X	N	X	N	X	N
caupolican	X	N	Y	N	X	N
bicoid	X	N	Y	Y	Y	Y
caudal	Y	Y	Y	Y	Y	Y
hunchback	Y	Y	Y	Y	Y	Y
Kruppel	X	N	Y	Y	Y	Y
giant	Y	Y	Y	Y	Y	Y
tailless	Y	Y	Y	Y	X	N
huckebein	Y	Y	Y	Y	X	N
even skipped	Y	N	Y	Y	Y	Y
odd skipped	Y	Y	Y	Y	Y	Y
runt	Y	N	Y	Y	Y	Y
fushi tarazu	Y	Y	Y	Y	Y	Y
paired	Y	Y	X	N	Y	N
engrailed	Y	N	Y	Y	X	N
labial	X	N	Y	Y	X	N
proboscipedia	X	N	X	N	X	N
Deformed	Y	Y	Y	Y	X	N
Sex combs reduced	X	N	Y	Y	X	N
Antennapedia	Y	N	Y	Y	X	N
abdominal A	X	N	Y	Y	Y	Y
Abdominal B	X	N	Y	Y	X	N
Ultrabithorax	X	N	Y	Y	Y	N
vasa	Y	Y	Y	N	Y	N
piwi	Y	N	Y	Y	Y	Y
Dicer-1	Y	Y	Y	Y	Y	N
oskar	N	N	Y	Y	Y	N
tudor	Y	Y	Y	Y	Y	Y
nanos	Y	Y	Y	Y	Y	Y
decapentaplegic	Y	N	Y	Y	Y	Y
bagpipe	X	X	Y	X	X	X
tinman	X	N	X	N	X	N
sloppy paired 1	Y	Y	Y	Y	Y	Y
wingless	Y	Y	Y	Y	Y	Y
midline	N	N	N	Y	N	N
Myocyte enhancer factor 2	Y	Y	Y	Y	Y	Y
doublesex	N	N	Y	Y	Y	Y
sisterless A	N	N	Y	Y	X	N
Sex lethal	Y	Y	Y	Y	Y	N
transformer	N	Y	N	Y	N	Y
deadpan	Y	Y	Y	Y	X	N

Table S5 (contd.)

	C. albipunctata		*M. abdita*		*E. balteatus*	
	Blast Search	Name Search	Blast Search	Name Search	Blast Search	Name Search
extra macrochaetae	Y	N	Y	Y	Y	N
outstretched	N	N	N	N	Y	N
brinker	Y	N	Y	Y	Y	N
dorsal	Y	Y	Y	N	Y	N
CrebA	Y	Y	Y	N	Y	N
C-terminal Binding Protein	Y	N	Y	N	Y	N
Medea	Y	N	Y	Y	Y	Y
nejire	Y	N	Y	Y	Y	Y
pannier	X	N	Y	Y	X	N
schnurri	Y	Y	Y	Y	Y	N
single-minded	X	N	Y	Y	X	N
snail	X	N	Y	Y	Y	Y
twist	Y	N	Y	N	Y	Y
zerknullt	Y	Y	Y	Y	Y	Y
pointed	Y	Y	Y	Y	Y	Y
rhomboid	Y	Y	Y	Y	Y	Y
spitz	Y	N	Y	N	Y	N
Star	Y	N	Y	Y	Y	Y
folded gastrulation	N	N	X	N	Y	N
screw	X	N	X	N	X	N
short gastrulation	Y	Y	Y	Y	Y	Y
twisted gastrulation	Y	N	Y	N	Y	Y
tolloid	Y	Y	Y	Y	Y	Y
tolloid related-1	Y	N	Y	N	Y	N
punt	Y	N	Y	N	Y	N
saxophone	Y	Y	Y	Y	Y	Y
thick vein	Y	N	Y	N	Y	N
Mothers against dpp	Y	Y	Y	Y	Y	Y
ventral nervous system defective	X	N	Y	Y	Y	Y
Merlin	Y	Y	Y	Y	Y	Y
expanded	Y	Y	Y	Y	Y	Y
hippo	Y	N	Y	N	Y	N
salvador	Y	N	Y	N	Y	Y
mob as tumor suppressor	Y	N	Y	N	Y	N
warts	Y	Y	Y	N	Y	Y
yorkie	Y	N	Y	N	Y	Y
hopscotch	Y	N	Y	Y	X	N
unpaired 2	N	N	N	N	X	N
domeless	Y	N	Y	N	Y	N
Socs36E	Y	N	Y	N	Y	N
Socs44A	Y	N	Y	Y	X	N
Socs16D	Y	N	Y	Y	Y	Y
Stat92E	Y	N	Y	Y	Y	Y
mex-3	Y	Y	Y	N	Y	N
orthodenticle	X	N	Y	Y	Y	Y
castor	N	N	Y	N	N	N
nubbin	Y	Y	Y	Y	Y	N
sichel	N	N	N	N	N	N

Y search and reciprocal blast positive
N search negative
X search positive and reciprocal blast negative

Section S2.2: Verification of Alternative Transcripts

Both Newbler and Trinity assemblers provide information on alternative transcripts. Previous work demonstrated that Newbler shows a low rate of false positive prediction of alternative transcripts, but fails to predict the complete set of isoforms identified by RT-PCR (Ewen-Campen et al. 2011; BMC Genomics 12: 61). No equivalent evidence is available for Trinity. Therefore, we tested the accuracy of alternative splicing predictions for Trinity on combined 454/Illumina data, and for Newbler on 454 data alone.

We selected 10 transcript clusters (Trinity) or isotigs (Newbler) each in *C. albipunctata* and *M. abdita,* which exhibit internal differences while sharing common flanking sequences, and tested them for alternative transcripts using RT-PCR (see Figure S7 and Table S6). The quality of prediction is similar across species, but varies between assembly methods. 50–60% of alternatively spliced transcripts predicted by Trinity, and 80% of those predicted by Newbler were detected by PCR. However, both methods miss a significant amount of additional bands, and the rate of false positives is quite high (20–50%). Again, Trinity does worse than Newbler in both of these cases. Considering that Trinity generally predicts a much larger number of alternative splice forms than Trinity (even if used on 454 data; see Table S6), we conclude that a large percentage of the predictions by Trinity are spurious or inaccurate. Therefore, 454 pyro-sequencing and Newbler assembly should be used if reliable predictions of alternative splicing events are required.

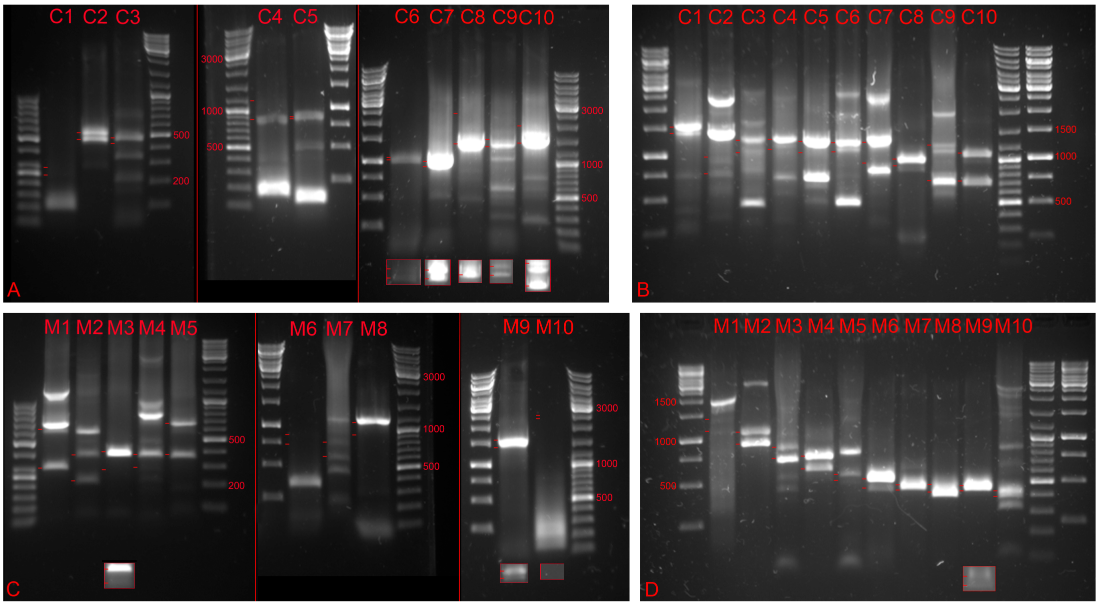

Figure S7: Verification of alternative transcript predictions. Agarose gels showing bands obtained with primer pairs to amplify two predicted alternative transcripts in each lane. (A) Test of the predictions for the *Clogmia* 454+Illumina dataset annotated with Trinity. (B) Test of the predictions for the *Clogmia* 454 dataset annotated with Newbler. (C) Test of the predictions for the *Megaselia* 454+Illumina dataset annotated with Trinity. (D) Test of the predictions for the *Megaselia* 454 dataset annotated with Newbler. Small horizontal lines show the putative positions of the predicted bands. In (A) and (C), PCR products have been run on gels with differing agarose concentrations (as indicated by vertical red lines). Insets in (A), (C), and (D) are taken from the same gels, using extended electrophoresis runs, or longer exposure times for higher resolution of bands.

Table S6: Verification of alternative transcript predictions. These tables summarize the results shown in Figure S7. (A) Results for Trinity (454/Illumina), (B) results for Newbler (454) assemblies. C stands for *Clogmia* isogroups, M for *Megaselia* isogroups. Y indicates presence of the expected band. N indicates absence of the expected band. Predicted band sizes are shown (in base pairs, bp). The number of extra bands (of different size than of those predicted) is shown in the corresponding column. Details on PCR conditions are given in the right-most column (polymerase used: BioMix Red, Bioline; Advantage 2 Polymerase Mix, Clontech; annealing temperature (^{o}C); and number of cycles c).

A

Trinity	Expected high band size	Expected high band present	Expected low band size	Expected low band present	extra bands	PCR details
C1	285	N	240	N	1	biomix; 50°C; 40c
C2	510	Y	460	Y	1	biomix; 50°C; 40c
C3	485	Y	420	N	4	biomix; 50°C; 40c
C4	1260	N	860	Y	1	biomix; 50°C; 40c
C5	890	Y	825	N	2	biomix; 50°C; 40c
C6	1100	Y	1050	Y	0	biomix; 50°C; 40c
C7	1000	Y	925	Y	1	advant; 50°C; 40c
C8	2520	N	1470	Y	2	advant; 50°C; 40c
C9	1640	Y	1480	Y	5	advant; 50°C; 40c
C10	2000	Y	1570	Y	4	advant; 50°C; 40c
M1	685	Y	300	Y	2	advant; 50°C; 40c
M2	400	Y	230	Y	4	advant; 50°C; 40c
M3	415	Y	300	N	1	advant; 50°C; 40c
M4	390	Y	330	Y	9	advant; 50°C; 40c
M5	680	Y	380	Y	3	advant; 50°C; 40c
M6	900	N	725	N	2	biomix; 50°C; 40c
M7	835	Y	635	Y	4	advant; 50°C; 40c
M8	1190	Y	940	N	1	biomix; 50°C; 40c
M9	1580	Y	1380	Y	0	biomix; 50°C; 40c
M10	2580	N	2380	N	1	biomix; 50°C; 40c

B

Newbler	Expected high band size	Expected high band present	Expected low band size	Expected low band present	extra bands	PCR details
C1	1680	Y	1500	Y	3	advant; 50°C; 40c
C2	1055	Y	795	Y	4	advant; 50°C; 40c
C3	1405	Y	1190	Y	4	advant; 50°C; 40c
C4	1370	Y	1235	Y	4	advant; 50°C; 40c
C5	1390	Y	1185	N	3	advant; 50°C; 40c
C6	1320	Y	1175	Y	4	advant; 50°C; 40c
C7	1370	Y	965	N	2	advant; 50°C; 40c
C8	1075	Y	945	Y	1	biomix; 50°C; 40c
C9	1295	Y	730	Y	3	advant; 50°C; 40c
C10	1105	Y	735	Y	1	biomix; 50°C; 35c
M1	1410	Y	1185	Y	3	advant; 50°C; 40c
M2	1105	Y	920	Y	1	biomix; 50°C; 35c
M3	890	Y	735	Y	2	advant; 50°C; 40c
M4	770	Y	640	Y	0	biomix; 50°C; 35c
M5	590	Y	520	Y	1	advant; 50°C; 40c
M6	535	Y	450	Y	0	biomix; 50°C; 40c
M7	500	Y	445	N	1	biomix; 45°C; 35c
M8	495	N	440	Y	0	biomix; 50°C; 40c
M9	490	Y	445	Y	0	biomix; 50°C; 40c
M10	450	Y	380	Y	8	advant; 50°C; 40c
