# Supplementary material for: Comparative transcriptomics of early dipteran development
Source: BMC Genomics. 2013 Feb 24;14:123. doi: 10.1186/1471-2164-14-123 (PMC3616871; doi:10.1186/1471-2164-14-123)
Supplement: Additional file 4 — Principal component analysis (PCA) of compositional bias. Contains Figure S8 showing the results of a PCA for amino acid distributions from concatenated sequences in all 21 species considered in our phylogenomic analysis. (PDF 112 kb) [file 1471-2164-14-123-S4.pdf]

Additional File 4:
Principal Component Analysis of Amino Acid Composition

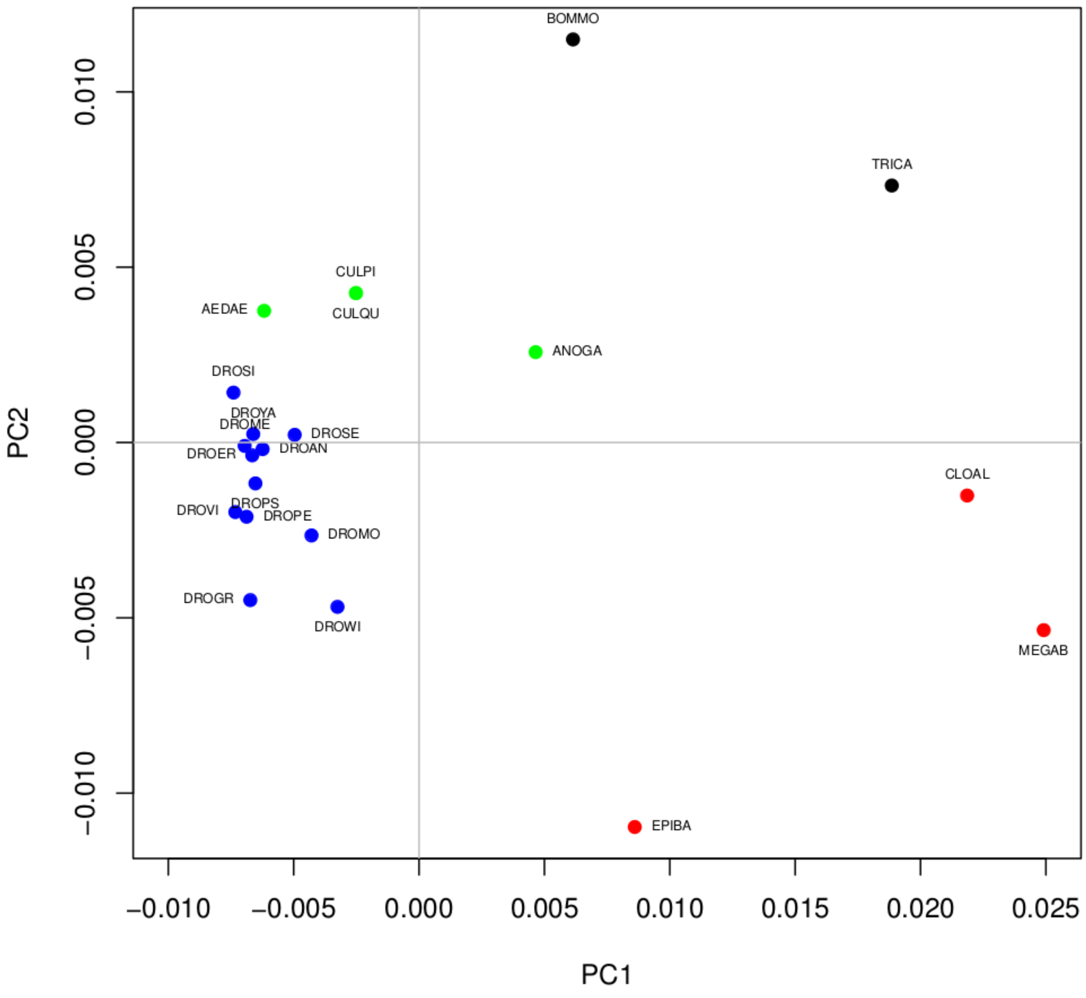

Figure S8. Principal Component Analysis (PCA) of amino acid composition. This figure shows the position of the concatenated sequences from the 21 species considered in our study along the two principal components (PCs) identified by our analysis. Transcriptome sequences of *C. albipunctata*, *M.* abdita, and *E. balteatus* are shown in red, outgroup sequences in black, mosquito species in green, and the drosophila clade in blue. Species abbreviations: CLOAL = *CLOgmia ALbipunctata,* etc. See Methods in main paper for details.
